# Generative Artificial Intelligence Models in Breast Cancer Diagnosis: A Systematic Review

**DOI:** 10.3390/diagnostics16203428

**Published:** 2026-10-21

**Authors:** Zainab Magaji Musa, Habeeba Adamu Kakudi

**Affiliations:** Department of Computer Science, Bayero University, Kano 700006, Nigeria; zmm2100015.pcs@buk.edu.ng

**Keywords:** generative artificial intelligence, deep learning, breast cancer diagnosis, systematic review, medical imaging, GAN, VAE, transformer

## Abstract

**Background**: This systematic review aims to evaluate and synthesize the current research on generative artificial intelligence (AI) models, focusing on their roles, performance, datasets, and evaluation metrics in improving breast cancer detection. **Method**: A systematic search using the PRISMA guideline was conducted. Four electronic databases (Science Direct, IEEE Explore, SpringerLink, and Scopus) and Google Scholar were searched using specific search strings for online articles published between 2020 and 2024. After applying specified inclusion and exclusion criteria, 91 articles were selected for data extraction, synthesis, and quality assessment. **Results**: This study reveals that generative AI models are used in breast cancer diagnosis for data augmentation, classification, and segmentation. For data augmentation, GAN has proven to be of highest effectiveness, while transformers have the highest effectiveness in terms of classification and segmentation. This review highlights the growing adoption of various publicly and privately available datasets, which have played a crucial role in training and validating these models. The findings showed the importance of selecting appropriate datasets and metrics to enhance the effectiveness of generative AI models in breast cancer diagnosis. A notable gap identified is the underrepresentation of African populations in the datasets used. Furthermore, the limited applicability of these models in real-world clinical settings is attributed to the lack of explainability. **Conclusions**: This review provides valuable insights into current practices, identifies existing gaps, and highlights areas for future research, aiming to improve the reliability and applicability of AI-based diagnosis in clinical settings.

## 1. Introduction

Breast cancer is a prevalent and lethal disease that remains one of the leading causes of mortality among women globally. The World Health Organization (WHO) asserts that abnormal cells in the breast proliferate uncontrollably, leading to tumors that may metastasize and become fatal if left untreated. In 2022, 2.3 million women were diagnosed with breast cancer worldwide, and 670,000 individuals succumbed to the disease. Post-adolescence, breast cancer can affect women of any age; however, its prevalence increases with advancing age. Health promotion and accurate diagnostic methods are fundamental in combating this disease, as early detection is vital for enhancing survival rates [1].

The emergence of deep learning and its effective use in breast cancer diagnosis has spurred a significant rise in research aimed at enhancing diagnostic precision. Developing effective and dependable deep learning techniques for forecasting the occurrence and advancement of cancer cells is essential for diminishing the worldwide breast cancer death rate [2]. To assist future researchers and practitioners in navigating this swiftly advancing subject, it is imperative to conduct a comprehensive evaluation and comparison of existing literature.

Numerous systematic literature studies concerning deep learning-based breast cancer detection have been published. Ref. [2] conducted a study of 13 empirical studies concerning breast cancer detection that employed diverse deep learning models across many imaging modalities. The scope was constrained by the limited quantity of articles examined. Ref. [3] broadened their analysis by reviewing 95 articles on genetic sequencing and histopathological imaging, emphasizing the insufficient application of attention processes in picture categorization tasks. Ref. [4] concentrated on CNN models and their hybrids, whereas [5] examined 80 publications that integrated deep learning and machine learning models, highlighting a deficiency in the utilization of models such as GAN, RNN, and LSTM for breast cancer prediction. Ref. [6] focused solely on deep learning applications in mammography.

A study of the existing systematic literature studies suggests that image modalities including MRI, ultrasound, and mammography are the main focus. Ref. [3] is among the few who have taken genetic sequencing and histology into account. However, their review, which encompassed 98 works from 2010 to 2021, is limited in light of the tremendous improvements in deep learning, particularly in generative AI, after 2021. Furthermore, the utilization of generative AI models was not specifically addressed in any of the current systematic literature studies. In recent years, generative AI models have garnered significant interest for their ability to generate novel data samples that closely resemble the characteristics of their training data. They are utilized in healthcare for diagnostics, drug discovery, and individualized treatment plans, among other applications.

This systematic review aims to deliver a thorough examination of generative AI models specifically utilized in breast cancer diagnosis. This study emphasizes empirical research employing generative models, including generative adversarial networks (GANs), Variational Autoencoders (VAEs), transformers, and recurrent neural networks (RNNs), across five diagnostic modalities: mammographic images, ultrasonographic images, MRI, histopathological images, and genetic sequencing and expression techniques. The study aims to evaluate the present state of research, identify recent achievements, and underscore significant gaps and future possibilities for the utilization of generative AI in breast cancer diagnosis.

### 1.1. Research Questions

The research questions answered are as follows:

1. What are the primary generative AI models utilized in breast cancer diagnosis, and how do they differ in their approaches and architectures?
2. Which generative AI models demonstrated the highest effectiveness in breast cancer detection, and what are the key factors contributing to their performance?
3. What datasets are commonly used in studies employing generative AI models for breast cancer diagnosis, and how do these datasets influence the training and performance of the models?
4. What evaluation metrics and methodologies are commonly used to assess the performance of generative AI models in breast cancer diagnosis, and how do they capture the effectiveness of the models?
5. What are the most common diagnostic modalities and the diagnostic accuracies of single and multiple modality approaches utilizing generative AI models in breast cancer diagnosis?
6. What are the current research gaps and challenges in the application of generative AI models for breast cancer detection, and what future directions are suggested by existing literature to address these gaps?

### 1.2. Cancer Diagnosis

Breast cancer represents a considerable risk to women’s physical and emotional well-being globally, with both morbidity and mortality rates exhibiting a marked rise. Ref. [7] emphasizes that patients diagnosed at an early stage exhibit a much higher survival rate than those found at later stages. Numerous diagnostic methods have been established, predominantly based on imaging and molecular biotechnology. These methodologies have been essential in the screening and confirmation of breast cancer [7,8,9]. This study examines only five methods: mammography, ultrasonography, MRI, histology, and genetic testing.

Mammography is a specialist imaging modality that use low-dose X-rays to assess the breast. Mammography is the predominant screening technique for breast cancer, proficient in identifying early indicators of the disease, frequently before the onset of symptoms. There are two categories: digital mammography, which employs digital receptors and computers for enhanced examination of breast tissue, and 3D mammography (tomosynthesis), which generates a three-dimensional image of the breast by capturing multiple X-ray images from various angles [7,8,9].

Ultrasonography employs high-frequency sound waves to produce intricate pictures of breast tissue. It is frequently utilized as an adjunct method to mammography, especially for women with dense breast tissue, where mammograms may exhibit diminished efficacy. It distinguishes between solid lumps and cysts containing fluid. It is contingent upon the operator and may overlook tiny tumors detectable by mammography [7,8,9].

Magnetic Resonance Imaging (MRI) uses powerful magnetic fields and radio waves to produce intricate images of the breast. MRI is very beneficial for high-risk individuals, determining the degree of malignancy and detecting abnormalities seen in mammography or ultrasound. It possesses elevated sensitivity, advantageous in thick breast tissue and in assessing the degree of malignancy. However, it exhibits elevated costs, restricted accessibility, and may produce false positives, resulting in unwarranted biopsies [7,8,9].

Histopathology entails the microscopic analysis of breast tissue specimens acquired via biopsy. Pathologists examine these specimens to detect malignant cells and furnish comprehensive information regarding tumor attributes. A biopsy entails the extraction of a tiny specimen of breast tissue for laboratory examination to conclusively detect cancer [7,8,9]. Multiple biopsy techniques exist:

7. Fine-Needle Aspiration (FNA) employs a slender needle to obtain tissue or fluid samples.
8. Core Needle Biopsy employs a bigger needle to extract a tiny cylindrical specimen of tissue.
9. Surgical biopsy entails excising a substantial portion of tissue or the complete mass.

Genetic testing examines the genetic material of cancer cells to identify the exact type and characteristics of breast cancer, which can affect treatment options [7,8,9].

10. HER2 Testing assesses the presence of elevated quantities of the HER2 protein in cancer cells, which facilitates their proliferation.
11. BRCA1 and BRCA2 Testing detects abnormalities in these genes that markedly elevate the risk of breast cancer.

### 1.3. Background of Generative AI Models

Generative AI includes deep learning models that analyze raw data and generate statistically plausible outputs in response to cues. These models acquire a simplified representation of their training data and employ it to produce new outputs that are analogous, yet not identical, to the original data [2]. Generative AI concentrates on developing models capable of producing novel and original content. These models possess the capability to independently generate data in the form of images, text, audio, or other information types. The primary idea behind generative AI is to enable robots to demonstrate creativity and develop material that is not explicitly coded into them [3]. Generative AI models leverage neural networks to recognize patterns and structures within existing data, enabling them to create new and original information. These models can be trained using several learning methodologies, including unsupervised and semi-supervised learning [10].

Over time, generative AI has become crucial in defining how we engage with technology. Picture it as the transition from AI systems only analyzing and processing data to actively generating something fresh. It is this creative leap that has opened doors to applications we would not have envisioned previously, making it a vital force in the technological world [11]. Experts have classed generative AI models into the following:

#### 1.3.1. Generative Adversarial Networks (GANs)

Generative adversarial networks (GANs) facilitate deep representation learning without necessitating extensively annotated training data. This is achieved via a competitive mechanism involving two neural networks [10]. The generator produces new data samples, whereas the discriminator distinguishes between authentic data from the domain and artificially generated content. As training advances, both networks consistently enhance their functionalities: the generator optimizes its output, while the discriminator gets increasingly proficient at differentiating actual from fake data. This iterative process persists until the produced content is almost indistinguishable from authentic data [12]. Generative adversarial networks (GANs) possess diverse applications, encompassing picture synthesis, semantic image editing, style transfer, image super-resolution, and classification [13].

#### 1.3.2. Variational Autoencoders (VAEs)

Variational Autoencoders (VAEs) comprise two neural networks, referred to as the encoder and decoder. The encoder converts input data into a concise, dense representation that preserves crucial information while eliminating extraneous features. The decoder subsequently reconstructs the original data from the compressed representation, allowing the model to acquire an efficient latent space. This enables users to sample new latent representations, which the decoder can utilize to produce unique data [14]. Variational Autoencoders (VAEs) have emerged as a prevalent method for the unsupervised learning of intricate data distributions. Their success is rooted in standard neural network topologies, enabling compatibility with training by stochastic gradient descent. They have exhibited considerable capability in producing various forms of intricate data, including handwritten digits, human faces, home numbers, CIFAR images, physical scene models, segmentation tasks, and forecasting future frames from static images [2].

#### 1.3.3. Recurrent Neural Networks (RNNs)

A recurrent neural network (RNN) is a particular sort of neural network developed for processing sequential input, such as natural language and time-series data. It includes feedback connections that enable the network to integrate previous outputs as inputs with new data at every time step. This feedback mechanism allows the network to remember prior inputs, rendering it effective for processing sequences, hence its designation as a recurrent neural network [2,3]

Recurrent neural networks (RNNs) are advantageous for generative tasks, as they can forecast the subsequent element in a sequence based on preceding items. Nonetheless, they encounter difficulties in producing extended sequences because of the vanishing gradient issue. To address this issue, sophisticated variations such Long Short-Term Memory (LSTM) and Gated Recurrent Unit (GRU) have been created Recurrent neural networks (RNNs) are extensively utilized in applications such as Apple’s Siri and Google’s voice search due to their capability to preserve historical information via internal memory. This skill renders them advantageous for activities such stock price forecasting, text synthesis, transcription, and machine translation [15,16].

#### 1.3.4. Transformer-Based Models

The transformer model has emerged as a prevalent deep learning architecture in multiple fields, including natural language processing (NLP), computer vision (CV), and speech processing. Originally developed as a sequence-to-sequence model for machine translation, transformer-based pre-trained models (PTMs) have subsequently exhibited superior performance across several applications, establishing the transformer architecture as the preferred choice in natural language processing, especially for PTMs [17].

Introduced by ref [11], the transformer model has swiftly transformed the domain of natural language processing. Models such as GPT and BERT, founded on this design, have markedly surpassed prior state-of-the-art models. The performance enhancement was so significant that contemporary advanced models predominantly depend on transformer-based designs [18]. Transformers, akin to recurrent neural networks, are engineered to handle sequential data; however, they accomplish this without necessitating sequential processing [11].

Two fundamental factors that render transformers exceptionally successful for text-based generative AI tasks are self-attention and positional encodings. Self-attention enables the model to allocate weights to various segments of the input, signifying their significance to the overarching context. Positional encoding, conversely, aids the model in comprehending the sequence of words in the input [11,19].

A transformer consists of many transformer blocks or layers, which incorporate self-attention layers, feed-forward layers, and normalization layers. These components collaboratively function to analyze and forecast sequences of tokenized data, encompassing text, protein sequences, or image patches [11,20].

## 2. Method

This research adheres to the Preferred Reporting Items for Systematic Reviews and Meta-Analyses (PRISMA) guidelines for systematic reviews [21]. PRISMA ensures a transparent and systematic process for locating, selecting, and critically reviewing existing literature. It also gives organized assistance for recognizing and evaluating relevant studies. In conformity with PRISMA criteria, the study protocol was registered in the International Prospective Register of Systematic Reviews (PROSPERO) under the registration number CRD42024566030.

### 2.1. Eligibility Criteria

The articles selected have a one-to-one mapping with the inclusion criteria of this review. To give current state-of-the-art knowledge on breast cancer diagnosis, articles published from 2020 to May 2024 were considered. The inclusion and exclusion criteria of this systematic review are stated in Table 1.

### 2.2. Search Strategy

The systematic search executed in this study was finalized in May 2024 to select the most pertinent studies that could assist in resolving the research issues. The authors meticulously organized and executed the selection of articles, reading, data extraction, and synthesis procedures in a systematic manner. The systematic literature review concentrated on credible scientific databases available to the authors. These include Science Direct, IEEE Xplore, SpringerLink, Scopus, and Google Scholar.

The search string comprises keywords and their synonyms linked by Boolean operators. The keywords were derived from the title and aims of the review. The search terms employed were (“generative AI” OR “GAN” OR “VAE” OR “Transformer” OR “RNN”) AND (“Diagnos*” OR “Detect*” OR “predict*”) AND (“Breast Cancer”). The search filters were configured to get publications published from 2020 to 2024.

### 2.3. Selection Process

The authors devised the aforementioned incremental approach to achieve the review’s objectives. The initial phase entailed locating and selecting pertinent papers for inclusion in the review. The subsequent phase entailed the elimination of duplicate entries from the selected articles; the residual articles were assessed for eligibility based on their titles and abstracts. The procedure was streamlined through the utilization of Rayyan Ai, an online platform for systematic review management. The complete texts of the chosen articles were thereafter evaluated according to the inclusion and exclusion criteria to ascertain their eligibility. Potentially pertinent papers were thoroughly reviewed, and data were extracted into a standardized format for quality evaluation and data synthesis.

To ensure thoroughness and decrease the chance of missing vital information, the reviewers separately undertook the study selection procedure. This independent evaluation allowed for varied perspectives and minimized bias. The use of duplicate evaluations boosted the reliability of the review by minimizing the odds of discarding important research, especially those requiring complicated judgments. All selected articles were saved in a single folder and imported onto Mendeley for easier and standard reference and citation.

### 2.4. Data Extraction

The extracted information from all included studies encompasses the following elements: reference and year of publication, the generative AI model employed, the specific generative AI technique proposed by the authors, the task for which the model was utilized in the breast cancer diagnosis process, the methodology implemented by the authors to achieve their objectives, the datasets used for training and validating the proposed model, the evaluation metrics applied to assess the model’s performance and their respective scores, and the diagnostic modalities employed to determine the presence of cancer.

### 2.5. Data Synthesis

The gathered data were synthesized for a comprehensive discussion. This entailed addressing each study issue using tables that encapsulate the principal findings, techniques, and performance measures from the studies presented. The utilized generative AI model, the proposed specialized models, and the tasks to which these models were applied in breast cancer diagnosis were assessed. The training and validation datasets, along with the assessment criteria and their corresponding scores, were analyzed. By collecting this information into tables, a clear and complete overview that allowed for an in-depth comparison and understanding of the effectiveness of different generative models was produced. This rigorous approach ensured that each research question was thoroughly addressed with well-structured and reliable data.

### 2.6. Quality Assessment

No standardized tool exists specifically for evaluating deep learning models, particularly generative models, in breast cancer diagnosis. Consequently, existing instruments were modified and integrated to develop a thorough quality assessment tool specifically designed for the distinctive characteristics of generative AI research. PROBAST, QUADAS-2, and other pertinent checklists were tailored to meet the requirements of this investigation. PROBAST (Prediction model Risk of Bias Assessment Tool) is utilized for evaluating the risk of bias (ROB) and applicability of studies concerning diagnostic and prognostic prediction models [22]. QUADAS-2 is intended to evaluate the quality of primary studies on diagnostic accuracy [23]. The integration of these robust tools guarantees a comprehensive and transparent quality evaluation, aiding in the synthesis and interpretation of the results in this systematic review. A total of 12 criteria were established, each assigned one of the following scores: Yes (indicating the criterion is met), No (indicating the criterion is not met), and Partial (indicating the criterion is partially met). “Yes” is assigned 2 points, “partially” is assigned 1 point, and “No” is assigned 0 points. The reviewers independently evaluated each study using 12 questions and assigned scores accordingly. The scores for each study were subsequently aggregated as the “Total Score.” The “maximum possible score” is calculated as

maximum possible score = 2×Number of Questions.

Percentage score is computed by the formula

$$\mathrm{Percentage}\;\mathrm{Score}=\frac{Total\;Score}{Maximum\;Possible\;Score}\times 100\%$$

The quality of each study was assessed using a percentage score categorized as follows: high quality: 80–100%, moderate quality: 60–79%, and low quality: below 60%. All rating inconsistencies were deliberated and reconciled among the writers to get a consensus. All scores and rationales for each rating were documented.

## 3. Result

A total of 1700 items were identified from the chosen databases. Subsequent to the elimination of duplicates, 1265 publications were evaluated based on their titles and abstracts. As a result, 765 articles were eliminated, resulting in 386 for comprehensive evaluation. Access to the complete text was unavailable for 298 articles; hence, only 94 articles were fully acquired. Following a comprehensive review of the texts, 29 were removed for failing to meet the inclusion criteria. Consequently, 65 studies retrieved from the databases were deemed eligible for inclusion in the systematic review.

A total of 986 were identified via Google Scholar. Following the title screening, 728 articles were removed for lack of relevance. Out of 258 studies selected for retrieval, 178 were discarded due to mismatched abstracts. As a result, 46 articles were eliminated owing to lack of access to the full text, and an additional 8 were removed for failing to meet the inclusion criteria. Consequently, 26 studies sourced from Google Scholar were deemed appropriate for inclusion in the systematic review. A total of 91 studies were ultimately included for synthesis in the study. The data obtained from the 91 papers included in this review were synthesized into several tables created while addressing each research issue independently. Figure 1 illustrates a PRISMA flow diagram detailing the study selection process.

The number of included articles in each of the selected years is shown in Figure 2. Year 2023 recorded the highest inclusion of generative AI models in breast cancer diagnosis. However, it is expected that before the end of year 2024, more research will be published in this area. This is because from January 2024 to May 2024, we found 21 articles that passed the inclusion criteria, which is higher than the number of articles in 2020, 2021, and 2022. Thus, generative AI models are being increasingly applied day by day.

As stated in the PRISMA flow diagram, the articles selected were obtained from different databases, including Science Direct, Scopus, IEEE Explore, SpringerLink, and Google Scholar. Figure 3 shows a chart of the details.

### 3.1. Quality of Studies

The 12 criteria that govern the methodological quality assessment are described with variable names as follows:

C1: Are the objectives and hypotheses of the study clearly stated?

C2: Is the study design appropriate for addressing the research questions?

C3: Is the source of the data clearly described and appropriate for the study?

C4: Are the data preprocessing steps adequately described and justified?

C5: Is the architecture and methodology of the deep learning model described in sufficient detail?

C6: Are the methods for training and validating the model adequately described, including the use of training, validation, and test sets?

C7: Are relevant performance metrics reported comprehensively?

C8: Is there a comparison with baseline models or existing methods to contextualize the performance of the proposed model?

C9: Are potential sources of bias identified, discussed, and addressed in the study?

C10: Is the methodology detailed enough to allow replication, including descriptions of the algorithms, parameters, and hardware used?

C11: Are the results clearly presented, statistically sound, and well-supported by the data?

C12: Are the findings interpreted in the context of clinical relevance, discussing the potential impact on clinical practice?

The result of the quality assessment shows a score variation between 17 to 23. Six studies achieved a score of 17, ten studies achieved a score of 18, two studies achieved a score of 19, eighteen studies achieved a score of 20, thirty-nine studies achieved a score of 21, four studies achieved a score of 22, and twelve studies achieved a score of 23. The quality of the included studies fell into two categories. Eighteen studies were of moderate quality, while the remaining seventy-three studies were of high quality. Details are shown in Table 2 where ✓ stands for “YES”, X stands for No and P stands for Partially.

### 3.2. Discussion

The selected studies were synthesized by organizing the findings around each research question. Tables were used to summarize and compare data from various studies, allowing for clear, structured answers to each research question. With this approach, the key aspects of the topic were systematically addressed, enhancing the clarity and comprehensiveness of the review.

#### 3.2.1. RQ1: What Are the Primary Generative AI Models Utilized in Breast Cancer Diagnosis, and How Do They Differ in Their Approaches and Architectures?

The primary generative AI models utilized in the process of breast cancer diagnosis included GAN, VAEs, RNN, and transformers. This study finds that these models were applied in the process of diagnosing breast cancers for data augmentation, segmentation, and classification.

##### Data Augmentation

Augmentation is a method employed to create fresh sample images from pre-existing ones [12]. Conventional augmentation techniques employ transformation functions, like rotation and cropping, to enhance dataset variety from original photos. Nonetheless, these techniques frequently generate implausible images and may result in superfluous data duplication within the dataset [10].

Deep learning is essential in medical imaging and clinical applications; nevertheless, its efficacy may be compromised by issues such as data scarcity, class imbalance in medical image datasets, and the production of unrealistic medical pictures by traditional augmentation methods. To tackle these issues, it is imperative to generate synthetic medical data that accurately mimics real images while maintaining therapeutically significant characteristics [10,24,25].

To tackle this issue, researchers investigate the application of generative AI models for the creation of synthetic medical data. Generative adversarial networks (GANs) and Variational Autoencoders (VAEs) are predominantly employed for this objective [10]. Various researchers have created distinct forms of GAN and VAE models to produce synthetic data, aiming to eliminate the issue of class imbalance in datasets for breast cancer diagnosis. The efficacy of these GAN-based models has been evaluated using diverse performance criteria to determine the enhanced classification or segmentation results obtained from the enriched data generated by GANs. Table 3 presents a detailed analysis of the studies that introduced several iterations of generative AI-based models for data augmentation.

##### Classification

Classification plays a crucial role in breast cancer diagnosis, enabling the categorization of medical images into different classes such as benign or malignant. Among the primary generative AI models, this review found that transformers and recurrent neural networks (RNNs) have shown significant potential in improving the accuracy and reliability of classification tasks. Transformers were initially designed for natural language processing tasks but have been adapted to handle image data through architectures like vision transformers (ViTs). The effective performance of vision transformers led researchers to develop different variants to solve different problems, especially in medical imaging [30,31,41]. Their ability to capture complex relationships within data makes them particularly effective for classification in breast cancer diagnosis.

Ref. [30] employed various vision transformer models such as Pooling-based Vision Transformer (PiT), Convolutional Vision Transformer (CvT), and Separable Vision Transformer (SepViT) for breast cancer classification. These models leverage self-attention mechanisms to process image patches, capturing intricate details that enhance classification accuracy. Ref. [7] developed a Hybrid CNN-Transformer network (HCTNet) that combines convolutional layers for feature extraction with transformer layers for detailed analysis. This hybrid approach improves the model’s ability to classify complex breast cancer images accurately. Ref. [39] proposed the Holistic Attention Network (HATNet) transformer, which focuses on integrating global contextual information for more accurate breast cancer classification.

RNNs, particularly their advanced variants like Long Short-Term Memory (LSTM) networks and Gated Recurrent Units (GRUs), are good at handling sequential data. In the context of breast cancer diagnosis, RNNs are useful for analyzing temporal sequences of medical images, such as mammogram series, to detect subtle changes over time. Ref. [25] utilized RNNs in combination with WHO and SDM datasets for classification tasks, demonstrating the ability of RNNs to handle sequential imaging data effectively. Ref. [51] explored a combined approach using RNN-LSTM-GRU models to improve classification accuracy. This hybrid model leverages the strengths of each RNN variant to process complex image sequences. Ref. [47] implemented RNN-LSTM models alongside transformers for a comprehensive approach to classification. This combination ensures robust handling of both sequential dependencies and spatial relationships within the data.

In breast cancer diagnosis, both transformers and RNNs have proven to be powerful tools for classification tasks. Transformers’ ability to capture detailed spatial relationships within images and RNNs’ proficiency in handling sequential data complement each other, offering robust solutions for accurate and reliable breast cancer classification. The continued development and integration of these generative AI models hold promise for further advancements in medical diagnostics, ultimately leading to improved patient outcomes. Table 4 shows all the works in this review that developed and utilized variants of generative AI models for breast cancer classification.

##### Segmentation

Segmentation is a critical task in breast cancer diagnosis, involving the precise delineation of regions of interest (ROIs) such as tumors within medical images. Accurate segmentation helps in assessing the extent of cancer, planning treatment, and monitoring response to therapy [7,38]. According to the outcome of this review, GANs and transformers are used to improve segmentation accuracy in breast cancer diagnosis.

Transformers, known for their powerful self-attention mechanisms, have been adapted to handle image segmentation tasks effectively. They capture global contexts and intricate details within images, making them well-suited for segmenting complex structures like breast tumors. Ref. [38] developed a transformer-based neural network called TraBS (Transformer-based Breast Segmentation), which uses the self-attention mechanism to accurately segment breast tissue and tumors from mammograms. This model improves segmentation accuracy by effectively capturing both local and global features. Ref. [7] created the Hybrid CNN-Transformer network (HCTNet), combining convolutional layers for initial feature extraction with transformer layers for detailed segmentation of breast lesions. This hybrid approach enhances the model’s ability to delineate tumor boundaries precisely. Ref. [69] proposed the Semantic-aware Transformer (SaTransformer), designed to improve the segmentation of semantic regions within breast cancer images. This model integrates semantic information into the segmentation process, resulting in more accurate and meaningful segmentation results.

GANs, with their capability to generate high-quality synthetic data, are also employed for segmentation tasks. Ref. [42] utilized Conditional GANs for breast tumor segmentation. cGANs generate synthetic mammogram images that are used to augment the training dataset, improving the model’s ability to segment tumors accurately. Ref. [46] developed the Breast Tumor Segmentation cGAN (BTS-cGAN), which focuses on generating high-quality synthetic breast cancer images. The authors utilized a parallel dilated convolution (PDC) module to retain the features of various sizes of masses and to effectively extract information about the masses’ edges and interior texture. Details are shown in Table 5.

#### 3.2.2. RQ2: Which Generative AI Models Demonstrated the Highest Effectiveness in Breast Cancer Detection, and What Are the Key Factors Contributing to Their Performance?

In the context of data augmentation, GANs demonstrated superior efficacy compared to all generative models examined in this review. They have been extensively utilized for data augmentation in breast cancer detection across multiple modalities, including ultrasound, mammography, and histopathological imaging. Particular types of GANs, like Conditional GAN (CGAN) and Wasserstein GAN (WGAN), have been employed to augment the quality of breast ultrasound pictures and mammograms by efficiently diminishing noise, thus enhancing the precision of cancer detection. Variations, including Phased GAN (PGAN) and Deep Convolutional GAN (DCGAN), have been utilized for augmentation; anomaly detection; and synthesis of mammograms, ultrasound pictures, genomic data, and MRI scans, leading to improved classification performance. To evaluate the efficacy of generative models in data augmentation, the researchers utilized the generated dataset alongside the original limited dataset for segmentation or classification. In all instances, supplementary data derived from GANs or VAEs significantly enhanced classification and segmentation accuracy.

The GAN-based model created by [34] enhanced classification accuracy by 4.04%. Anwar et al. (2024) employed generative adversarial networks (GANs) to produce synthetic imaging data derived from a radiogenomic dataset [14]. This synthetic data, almost indistinguishable from authentic photos, was generated using a supervised neural network to improve breast cancer diagnosis. The research indicated that the incorporation of an autoencoder and dropout models into the proposed neural network resulted in a 1.8% enhancement in accuracy.

Ref. [35] examined a data imbalance in cancer gene expression datasets and proposed an improved deep learning-based Wasserstein Generative Adversarial Network (WGAN) model. This model offers a dependable training progress indicator and accurately reflects the intrinsic properties of the data. The WGAN mitigates data imbalances at the dataset level by generating additional samples from the minority class, leading to a 3% enhancement in accuracy.

Ref. [43] introduced innovative deep architectures termed AGAN, which integrate GAN and Variational Autoencoder systems for the enhancement of mammographic pictures. The newly generated image sets from the autoencoder-GAN model were utilized to train the classification model, resulting in a 12% enhancement in AUC.

Ref. [55] presented a DCGAN to address imbalanced data by synthesizing new images for the training dataset. Synthetic and genuine photos were subsequently identified with CNN and GoogleNet, which demonstrated superior performance in managing imbalanced data compared to traditional augmentation, achieving an accuracy increase of 1% to 4%.

Ref. [29] proposed an architecture that initiates the exclusion of pectoral muscles from the dataset, succeeded by data augmentation employing Variational Autoencoders (VAEs). U-Net and its derivatives were subsequently utilized for breast cancer categorization, yielding a 5.37% enhancement in accuracy.

Ref. [10] presented a Conditional Generative Adversarial Network (CGAN) designed to produce synthetic mammograms for various conditions, including normal, benign, and malignant cases. The produced samples were integrated into the MIAS dataset to rectify class imbalance. This enhancement increased the efficacy of both 2-class and 3-class breast cancer categorization by 3.9%.

The swift progress in generative artificial intelligence indicates that deep learning-based data synthesis can improve imaging quality, especially for inferior acquisition devices. It can also surmount the constraints of conventional imaging techniques by enhancing aspects like imaging velocity, resolution, modality, and slice staining procedures [78].

In terms of categorization, transformers have exhibited superior efficacy compared to the generative models analyzed in this study. A systematic review by [3] indicated that Convolutional Neural Networks (CNNs) are the most precise and prevalent models for breast cancer diagnosis, with accuracy metrics as the predominant evaluation approach. Nevertheless, CNNs encounter difficulties in capturing long-range relationships because of the concentrated characteristics of convolution operations. This constraint may hinder their capacity to effectively delineate and recognize related aspects of breast lesions, which are crucial for an exact diagnosis [60]. This constraint promotes the utilization of vision transformers, which are adaptations of the extremely effective transformers employed in natural language processing.

Vision transformers and their derivatives in breast cancer diagnosis have been directly compared to CNN-based models, demonstrating superior performance ratings. In the study by [39], the transformer-based model HATnet (accuracy = 0.70 ± 0.0024) outperforms the existing CNN model Y-Net (accuracy = 0.62 ± 0.0074).

Ref. [20] presented a transfer learning methodology utilizing vision transformers for the classification of breast masses in mammograms. Their model attained an area under the receiver operating characteristic (ROC) curve of 1 ± 0, exceeding the performance of CNN-based transfer learning models.

Ref. [64] similarly offered a transformer-based model assessed by a five-fold cross-validation technique. In contrast to traditional methods necessitating considerable preprocessing—such as optimal window cropping, removal of the chest wall or pectoral muscle, and registration of two-view images—their four-image (two-view, two-side) transformer model attained an AUC of 0.818 ± 0.039. This markedly surpassed the leading multi-view CNNs, which attained an AUC of 0.784 ± 0.016 (*p* = 0.009). The transformer model surpassed two one-view-two-side models, which recorded AUC values of 0.724 ± 0.013 for the CC view and 0.769 ± 0.036 for the MLO view.

Research combining a hybrid technique of transformers and CNNs has demonstrated superior performance compared to those utilizing either CNNs or transformers independently. Ref. [41] introduced a hybrid transformer-CNN model called the BTS-ST network, which outperformed existing transformer-based and CNN-based models, attaining superior results in F1 (0.856), precision (0.792), and AUC (0.813). Consequently, the amalgamation of CNNs and transformers presents significant potential for future progress. Table 5 presents the specifics of each work considered in this review.

Transformers have demonstrated significant efficacy in segmentation compared to the generative models analyzed in this analysis. Ref. [38] created a segmentation model named TraBS, attaining an accuracy of 0.864 ± 0.081. This model surpassed the CNN-based model nnUNet, which attained an accuracy of 0.824 ± 0.144 when evaluated on the identical datasets. This demonstrates the enhanced efficacy of transformer-based models in accurately collecting and segmenting intricate breast cancer imaging characteristics compared to conventional CNN models.

Furthermore, Ref. [7] presented a segmentation model called HCTNet, which enhanced the previously superior method, TransUNet. HCTNet exhibited improvements of 0.82%, 1.92%, 0.66%, and 0.99% in Dice, Jaccard, Accuracy, and Recall, respectively, and also displayed the lowest Hausdorff Distance (HD) value. These enhancements underscore the model’s capacity to deliver more accurate and dependable segmentation results.

Additionally, Ref. [41] introduced a hybrid transformer-CNN model called the BTS-ST network. This model attained superior performance relative to current transformer-based and CNN-based models, achieving scores of 0.908 in F1, 0.978 in AUC, and 6.8596 in HD95 metrics. The exceptional performance of the BTS-ST network highlights the efficacy of integrating transformers and CNNs to harness their advantages and attain more precise and resilient segmentation outcomes. The comprehensive overview of the included research is presented in Table 6.

#### 3.2.3. RQ3: What Datasets Are Commonly Used in Studies Employing Generative AI Models for Breast Cancer Diagnosis, and How Do These Datasets Influence the Training and Performance of the Models?

This section gives a detailed description of the datasets utilized by the included studies. The datasets provide the necessary input for training the models and evaluating their performance [30]. The quality and quantity of the datasets directly affect the accuracy and effectiveness of the models [57]. Thus, it is important to select appropriate datasets so as to achieve an effective result in the diagnosis of breast cancer using generative AI models [30,31,73].

Table 7 provides an overview of the datasets commonly utilized in breast cancer diagnosis research. The table contains the following columns: “Data Sets Utilized”, which lists the names of datasets used in the reviewed studies; “Description”, detailing the types of images, modalities, patient demographics, imaging methods, and classification labels in each dataset; “Accessibility”, indicating whether the dataset is publicly available or restricted; “Authors”, which includes researchers who employed the datasets in their studies; and “Frequency”, showing how often each dataset was used in the reviewed studies. The DDSM dataset was the most frequently used, appearing in 15 studies. The BUSI dataset followed, being utilized in 12 studies, while the BreakHis dataset was employed in 11 studies. The MIAS dataset appeared in eight studies, and the INbreast dataset was used in seven studies. The BACH dataset and Wisconsin dataset were each employed in six studies. Several datasets, such as the Breast Carcinoma Subtyping (BRACS) and Breast Invasive Carcinoma (BRCA) datasets were utilized in two studies, while others like AIDPATH, Breast biopsy dataset, Camelyon, and many more, were each used in only pme study, highlighting the diverse range of datasets explored in breast cancer diagnosis research.

#### 3.2.4. RQ4: What Evaluation Metrics Are Commonly Used to Assess the Performance of Generative AI Models in Breast Cancer Diagnosis, and How Do They Capture the Effectiveness of the Models?

Here, a detailed description of the evaluation metrics utilized by the included studies is given. Evaluation metrics quantify a model’s performance by measuring its accuracy, reliability, robustness, and relevance to the task, ensuring its effectiveness in achieving the desired outcomes [31,32,34]. The studies reviewed utilized metrics such as accuracy, recall, precision, F1 score, specificity, AUC-ROC, Matthew’s Correlation Coefficient (MCC), Dice Similarity Coefficient (DSC), Intersection over Union or Jaccard Similarity Coefficient (JSC), and Structural Similarity Index (SSIM).

Accuracy emerged as the most frequently used metric, appearing in 66 studies, highlighting its importance in capturing overall correctness of predictions. Recall (sensitivity)**,** crucial for identifying true positives, was employed in 39 studies, reflecting its value in clinical contexts where missing a positive case can have severe consequences. F1 score and precision were utilized in 24 studies each, balancing the trade-off between sensitivity and positive predictive value. Metrics like specificity (13 studies) and AUC-ROC (25 studies) were also widely adopted, providing complementary insights into model performance by evaluating true negative detection and overall discriminative ability, respectively. Lesser-used metrics included Matthew’s Correlation Coefficient (MCC) (three studies) and Kappa Score (two studies), often applied in imbalanced datasets to assess agreement and reliability. Spatial overlap metrics such as Dice Similarity Coefficient (DSC) and Intersection over Union (IoU) were specific to segmentation tasks, appearing in five and three studies, respectively. Image quality metrics like SSIM and DSSIM were less commonly used, featuring in four and one study(s), respectively, with their focus on structural and intensity-based image comparisons. Peak Signal-to-Noise Ratio (PSNR)**,** measuring image fidelity, was cited in two studies.

In Table 8, TP (True-Positive) indicates that the method correctly identified the disease as positive; FN (False-Negative) means the method incorrectly identified the disease as negative. TN (True-Negative) refers to the method correctly classifying the disease as negative, while FP (False-Positive) shows that the method incorrectly identified the disease as positive. μreal and μvirtual means the average of the real image and virtual image, respectively. σreal and σvirtual are the variance of the real image and virtual image, respectively. σreal_virtual stands for covariance of the real image and virtual image. C_1_ and C_2_ are constants.

#### 3.2.5. RQ5: What Are the Most Common Diagnostic Modalities and the Diagnostic Accuracies of Single and Multiple Modality Approaches Utilizing Generative AI Models in Breast Cancer Diagnosis

The diagnostic modality in breast cancer diagnosis is crucial as it determines the accuracy, efficiency, and early detection of the disease, directly influencing patient outcomes and treatment strategies [7,8,9]. Some studies utilized single modalities, while others employed multiple modalities.

Single modalities, such as mammography, ultrasonography, MRI, histopathology, and genomics, dominate the studies, with mammography being the most frequently employed, referenced in 37 studies, and demonstrating an accuracy range of 78% to 100%. Histopathology, considered the gold standard for definitive diagnosis, is highlighted in 25 studies with an accuracy range of 90% to 99%. Ultrasonography, cited in 13 studies, followed, showing an accuracy between 80% and 98%. Genomics, though cited only five times, achieved a high accuracy of up to 98%.

Multiple modality approaches, such as combining imaging with genomic data or integrating different imaging methods, are referenced in fewer studies but generally demonstrate enhanced diagnostic performance. For instance, the combination of imaging and genomics achieves an accuracy of up to 99%, reflecting the complementary strengths of these methods. The integration of ultrasonography and histopathology, reported in two studies, provides a robust diagnostic accuracy range of 91% to 99%. Overall, multiple modality approaches tend to outperform single modalities, emphasizing the potential for improved accuracy and reliability through integrated diagnostic strategies.

#### 3.2.6. RQ6: What Are the Current Research Gaps and Challenges in the Application of Generative AI Models for Breast Cancer Detection, and What Future Directions Are Suggested by Existing Literature to Address These Gaps?

##### Gaps Identified

12. The generative AI models are mostly applied separately for tasks like augmentation, classification, and segmentation in breast cancer diagnosis. There is limited integration of these models for a stronger model by combining their respective strengths.
13. The studies report high accuracy in model performance, but there is a gap in extensive clinical validation of these models. Robust clinical trials and validations are crucial to demonstrate real-world applicability and benefits in clinical settings.
14. Interpretability and explainability of AI-generated results are overlooked by the studies. Future research could focus on developing methods to explain how AI models arrive at their decisions to enhance trust and adoption by healthcare professionals
15. There are relatively few studies combining multiple modalities. These studies generally demonstrate higher diagnostic accuracies, suggesting potential benefits of multimodal approaches. Future research should explore more combinations and evaluate their synergistic effects on accuracy and clinical applicability.
16. Most datasets focus on static images rather than longitudinal data capturing disease progression over time. Integrating longitudinal data and multimodal imaging could improve model accuracy and clinical utility.
17. There is a lack of diversity in datasets concerning ethnic backgrounds and geographic regions. Models trained on homogeneous datasets may not generalize well to diverse populations, emphasizing the need for datasets that reflect global demographic variability.

##### Future Directions

Future directions aim to address current gaps and propel research in generative AI models for breast cancer diagnosis towards more robust, reliable, and clinically impactful applications, ultimately benefiting both patients and healthcare providers. Future researches should:

18. Consider hybrid models that combine the strengths of different generative models (e.g., GANs with transformers or RNNs) for more robust and efficient performance in breast cancer diagnosis tasks such as data augmentation, classification, and segmentation.
19. Conduct comprehensive clinical trials to validate the efficiency and reliability of generative AI models in real-world medical scenarios. This includes evaluating their impact on diagnostic accuracy, treatment planning, and patient outcomes.
20. Develop AI models that integrate information from longitudinal data, multiple imaging modalities (e.g., MRI, ultrasound, mammography), and other clinical data (genomic, pathology) for more comprehensive diagnosis and treatment planning.
21. Embrace explainable AI to make AI-driven diagnoses more transparent and understandable to healthcare providers, enhancing trust and adoption.
22. Implement federated learning approaches to train models across multiple institutions while preserving data privacy, enabling larger and more diverse datasets for model training.
23. Implement continual learning frameworks that allow AI models to continuously update and improve over time with new data and insights, ensuring adaptability to evolving medical knowledge.
24. Encourage international collaboration to collect diverse datasets that represent varied populations and healthcare systems, enhancing the robustness and generalizability of AI models for breast cancer diagnosis.
25. Foster collaboration between computer scientists, medical professionals, and bio-informaticians to ensure that generative AI models are developed with a deep understanding of clinical needs and challenges.

## 4. Conclusions

In conclusion, the integration of generative AI models presents a transformative opportunity for enhancing breast cancer detection accuracy and improving diagnostic outcomes. Our review highlights the significant potential of models such as GAN, VAEs, RNNs, and transformers and their variants in augmenting datasets and refining classification and segmentation tasks. While the reported accuracy rates are promising, the successful implementation of these models in clinical settings hinges on addressing existing gaps, including the need for diverse datasets and rigorous clinical validation.

Future research should prioritize the development of hybrid approaches that combine the strengths of various generative models alongside collaborative efforts between technology and healthcare professionals. By fostering interdisciplinary partnerships and conducting extensive clinical trials, we can ensure that these advanced models are not only effective in controlled environments but also applicable and reliable in real-world clinical practice. Ultimately, the successful application of generative AI in breast cancer detection has the potential to significantly enhance early diagnosis, improve patient outcomes, and reduce healthcare costs.

## Figures and Tables

**Figure 1:**
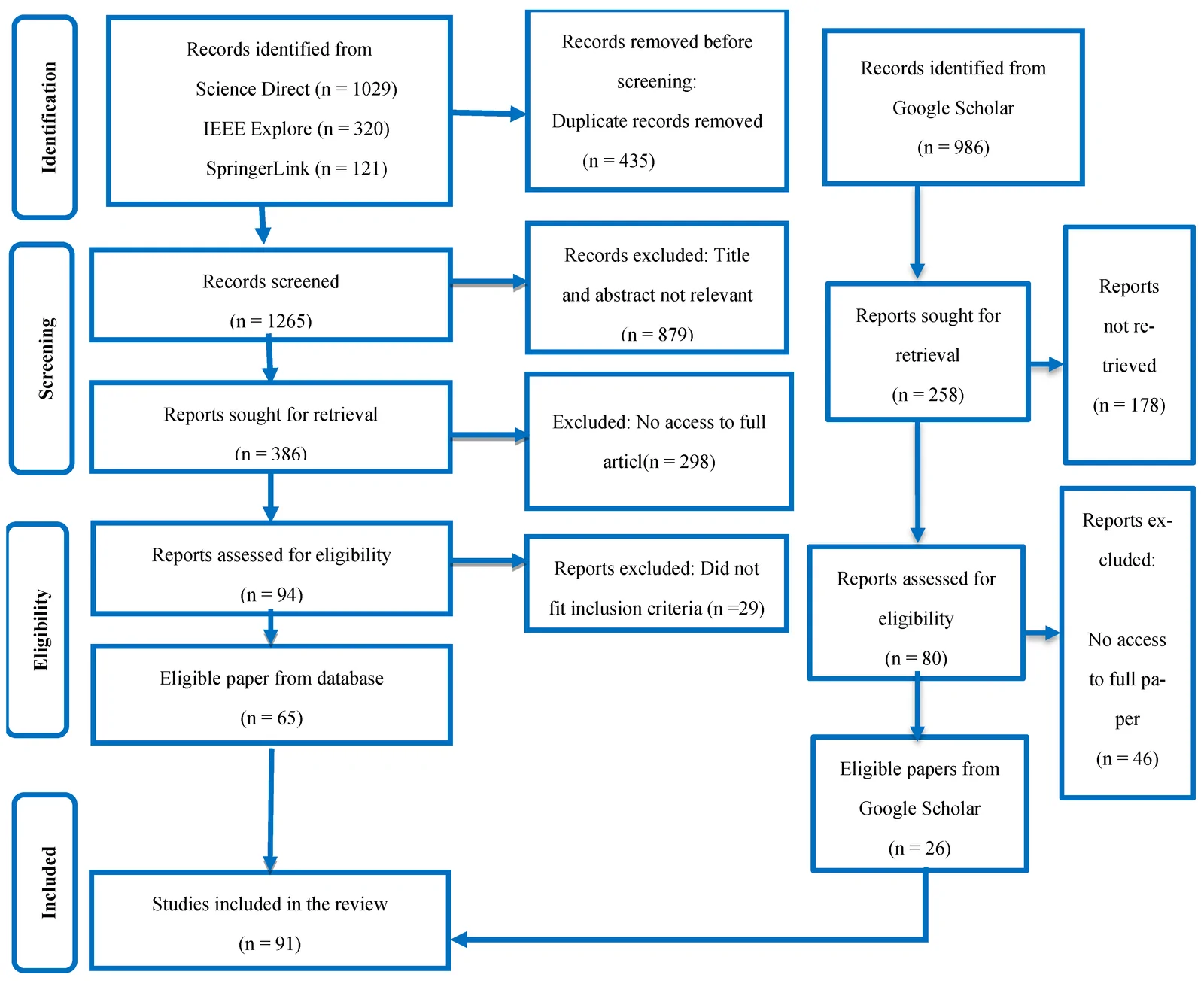
PRISMA flow diagram of the study selection process.

**Figure 2:**
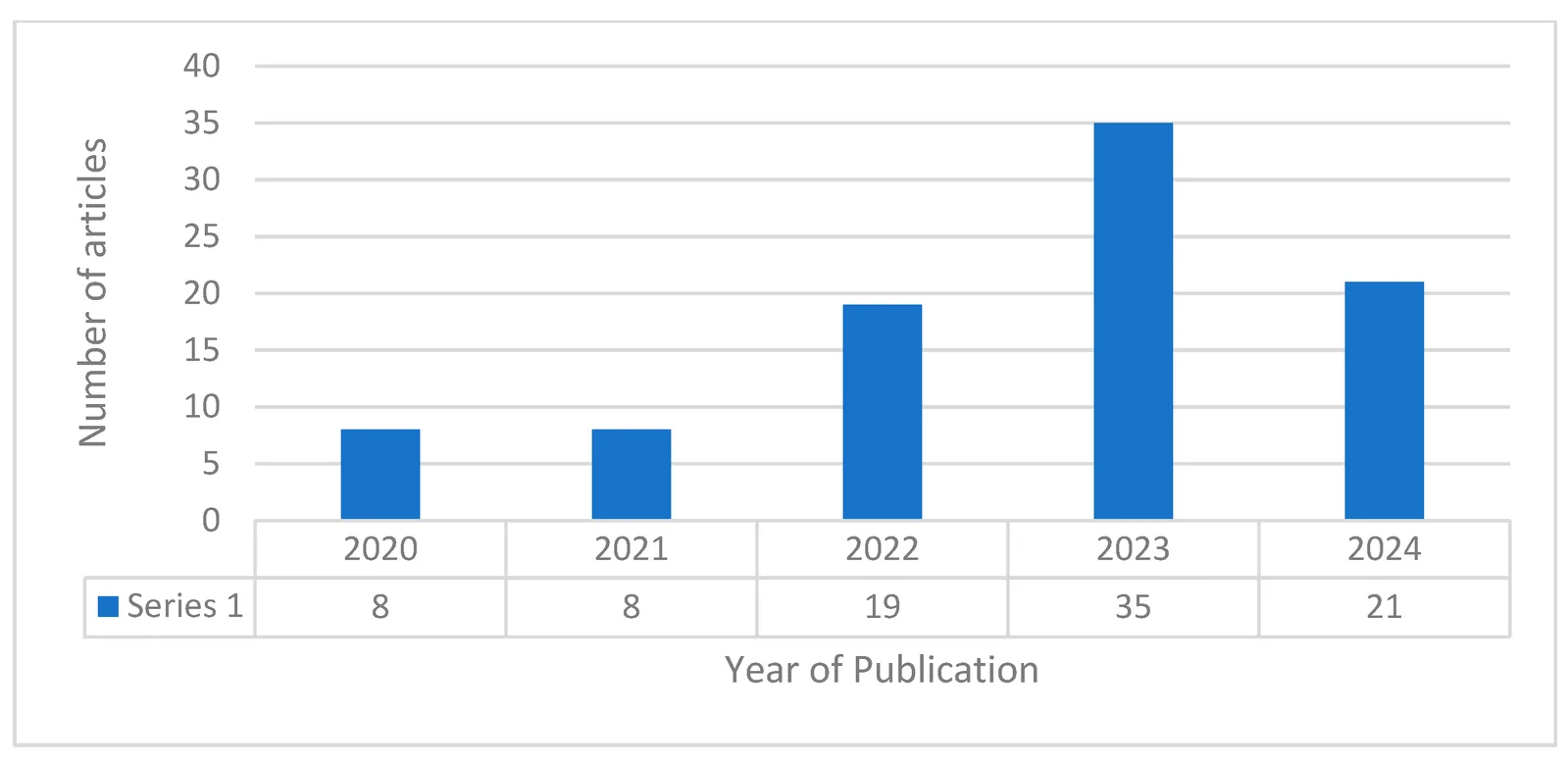
Number of publications per year.

**Figure 3:**
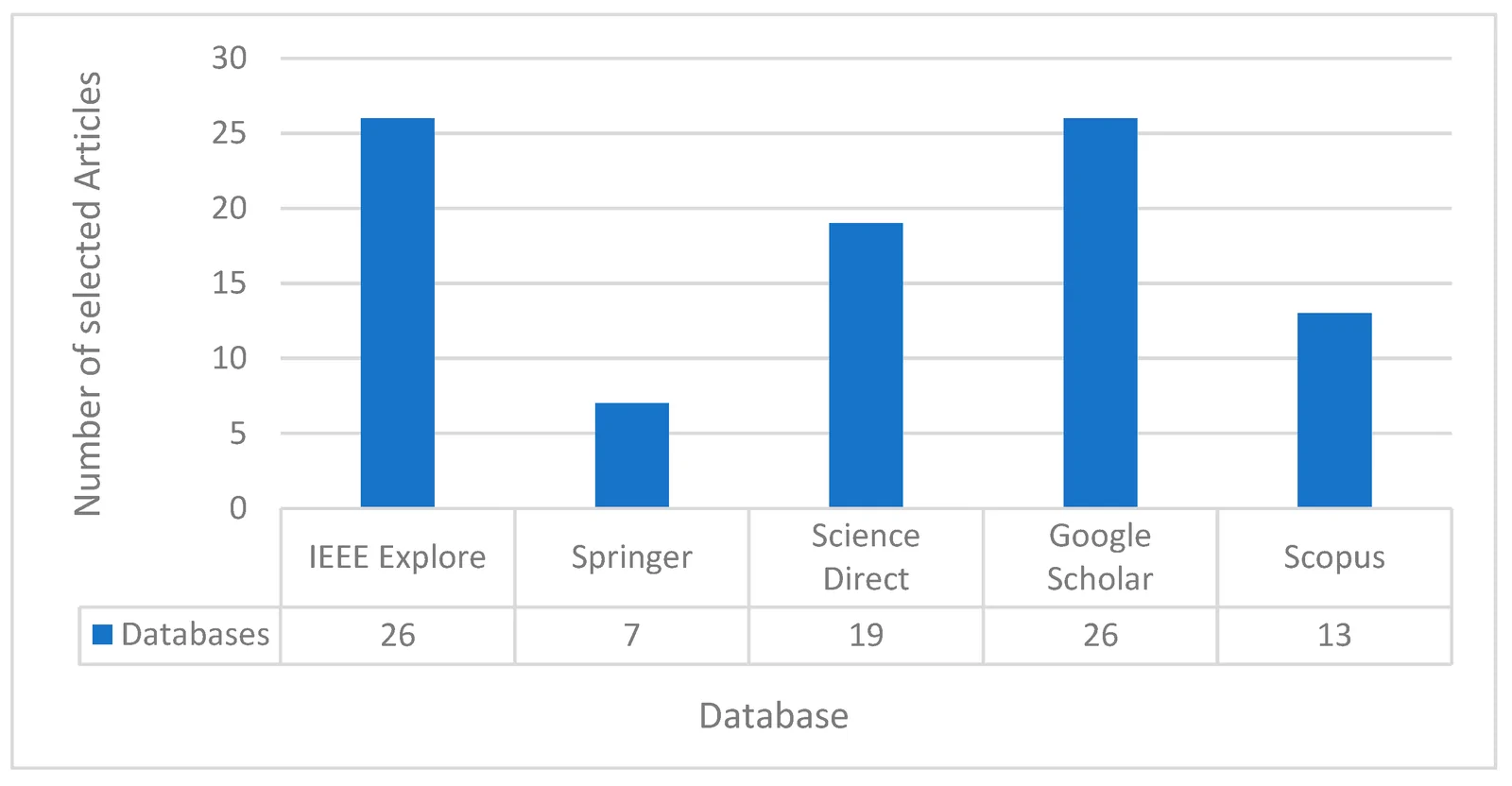
Breakdown of the articles’ databases.

**Table 1:** Criteria for inclusion and exclusion.

Inclusion	Exclusion
Studies published from 2020 to 2024	Studies earlier than 2020 were not included
Studies involving experimental results	Studies without experimental results were not selected
Studies that involved breast cancer	Studies that involved other cancers were not reviewed
Studies that work with generative AI models	Studies that work with models other than generative AI models were not included
Studies written in English only	Studies written in other languages were not considered
Only journal articles and conference papers were studied	Sources like books, reports, and dissertations were not included

**Table 2:** Quality assessment scores of the included studies.

Study	C1	C2	C3	C4	C5	C6	C7	C8	C9	C10	C11	C12	Tscore	%Score	Quality
[24]	✓	✓	✓	✓	✓	✓	✓	✓	P	✓	✓	P	22	91.7%	High Quality
[25]	✓	✓	✓	✓	✓	✓	✓	✓	X	✓	✓	P	21	87.5%	High Quality
[10]	✓	✓	✓	✓	✓	✓	✓	✓	X	✓	✓	P	21	87.5%	High Quality
[26]	✓	✓	✓	✓	✓	✓	✓	✓	X	✓	✓	P	21	87.5%	High Quality
[27]	✓	✓	✓	✓	✓	✓	✓	✓	X	✓	✓	P	21	87.5%	High Quality
[28]	✓	✓	✓	✓	✓	✓	P	✓	X	✓	✓	P	20	83.3%	High Quality
[12]	✓	✓	✓	✓	✓	✓	P	✓	✓	✓	✓	P	22	91.7%	High Quality
[29]	✓	✓	✓	✓	✓	✓	P	✓	X	✓	✓	P	20	83.3%	High Quality
[30]	✓	✓	✓	✓	✓	✓	✓	✓	X	✓	✓	P	21	87.5%	High Quality
[14]	✓	✓	✓	✓	✓	✓	P	✓	X	✓	✓	P	20	83.3%	High Quality
[13]	✓	✓	✓	✓	✓	✓	✓	✓	X	✓	✓	P	21	87.5%	High Quality
[31]	✓	✓	✓	✓	✓	✓	P	✓	X	✓	✓	P	20	83.3%	High Quality
[32]	✓	✓	✓	✓	✓	✓	P	✓	X	✓	✓	P	20	83.3%	High Quality
[33]	✓	✓	✓	✓	✓	✓	X	✓	X	✓	✓	P	19	79.1%	Moderate Quality
[34]	✓	✓	✓	✓	✓	✓	✓	✓	✓	✓	✓	P	23	95.8%	High Quality
[35]	✓	✓	✓	✓	✓	✓	P	✓	X	✓	✓	P	20	83.3%	High Quality
[36]	✓	✓	✓	✓	✓	✓	✓	✓	X	✓	✓	P	21	87.5%	High Quality
[37]	✓	✓	X	✓	✓	✓	✓	✓	X	✓	P	P	18	75.0%	Moderate Quality
[38]	✓	✓	✓	✓	✓	✓	✓	✓	X	✓	✓	P	21	87.5%	High Quality
[39]	✓	✓	✓	✓	✓	✓	✓	✓	X	✓	✓	P	21	87.5%	High Quality
[7]	✓	✓	✓	✓	✓	✓	✓	✓	✓	✓	✓	P	23	95.8%	High Quality
[40]	✓	✓	✓	✓	✓	✓	✓	✓	X	✓	✓	P	21	87.5%	High Quality
[41]	✓	✓	✓	✓	✓	✓	✓	✓	✓	✓	✓	P	23	95.8%	High Quality
[42]	✓	✓	✓	✓	✓	✓	P	✓	X	✓	✓	P	20	83.3%	High Quality
[43]	✓	✓	✓	✓	✓	✓	✓	✓	X	✓	✓	P	21	87.5%	High Quality
[44]	✓	✓	✓	✓	✓	✓	✓	✓	X	✓	✓	P	21	87.5%	High Quality
[45]	✓	✓	X	✓	✓	✓	✓	✓	X	✓	P	P	18	75.0%	Moderate Quality
[18]	✓	✓	X	✓	✓	✓	✓	✓	X	✓	P	P	18	75.0%	Moderate Quality
[46]	✓	✓	✓	✓	✓	✓	✓	✓	X	✓	✓	P	21	87.5%	High Quality
[47]	✓	✓	✓	✓	✓	✓	✓	✓	X	✓	✓	P	21	87.5%	High Quality
[48]	✓	✓	✓	✓	✓	✓	✓	✓	P	✓	✓	P	22	91.7%	High Quality
[49]	✓	✓	✓	✓	✓	✓	✓	✓	✓	✓	✓	P	23	95.8%	High Quality
[50]	✓	✓	✓	✓	✓	✓	X	✓	X	✓	P	P	18	75.0%	Moderate Quality
[51]	✓	✓	X	✓	✓	✓	P	✓	X	✓	✓	P	18	75.0%	Moderate Quality
[52]	✓	✓	✓	✓	✓	✓	✓	✓	X	✓	✓	P	21	87.5%	High Quality
[53]	✓	✓	✓	✓	✓	✓	P	✓	X	✓	✓	P	20	83.3%	High Quality
[54]	✓	✓	✓	✓	✓	✓	P	✓	X	✓	✓	P	20	83.3%	High Quality
[55]	✓	✓	✓	✓	✓	✓	✓	✓	✓	✓	✓	P	23	95.8%	High Quality
[56]	✓	✓	✓	✓	✓	✓	✓	✓	X	✓	✓	P	21	87.5%	High Quality
[57]	✓	✓	✓	✓	✓	✓	✓	✓	P	✓	✓	P	22	91.7%	High Quality
[58]	✓	✓	✓	✓	✓	✓	✓	✓	X	✓	✓	P	21	87.5%	High Quality
[59]	✓	✓	✓	✓	✓	✓	P	✓	P	✓	✓	P	21	87.5%	High Quality
[60]	✓	✓	✓	✓	✓	✓	P	✓	P	✓	✓	P	21	87.5%	High Quality
[61]	✓	✓	✓	✓	✓	✓	✓	✓	X	✓	✓	P	21	87.5%	High Quality
[62]	✓	✓	✓	✓	✓	✓	P	✓	X	✓	✓	P	20	83.3%	High Quality
[63]	✓	✓	X	✓	✓	✓	✓	✓	X	✓	P	P	18	75.0%	Moderate Quality
[64]	✓	✓	X	✓	✓	✓	P	✓	X	✓	P	P	17	70.8%	Moderate Quality
[19]	✓	✓	✓	✓	✓	✓	✓	✓	X	✓	✓	P	21	87.5%	High Quality
[65]	✓	✓	✓	✓	✓	✓	✓	✓	X	✓	✓	P	21	87.5%	High Quality
[20]	✓	✓	✓	✓	✓	✓	✓	✓	X	✓	✓	P	21	87.5%	High Quality
[66]	✓	✓	✓	✓	✓	✓	✓	✓	X	✓	✓	P	21	87.5%	High Quality
[67]	✓	✓	✓	✓	✓	✓	✓	✓	X	✓	✓	P	21	87.5%	High Quality
[68]	✓	✓	✓	✓	✓	✓	✓	✓	✓	✓	✓	P	23	95.8%	High Quality
[69]	✓	✓	✓	✓	✓	✓	✓	✓	X	✓	✓	P	21	87.5%	High Quality
[70]	✓	✓	✓	✓	✓	✓	✓	✓	X	✓	✓	P	21	87.5%	High Quality
[71]	✓	✓	✓	✓	✓	✓	✓	✓	X	✓	P	P	20	83.3%	High Quality
[72]	✓	✓	✓	✓	✓	✓	✓	✓	✓	✓	✓	P	23	95.8%	High Quality
[73]	✓	✓	✓	✓	✓	✓	P	✓	X	✓	✓	P	20	83.3%	High Quality
[74]	✓	✓	✓	✓	✓	✓	✓	✓	✓	✓	✓	P	23	95.8%	High Quality
[75]	✓	✓	✓	✓	✓	✓	✓	✓	X	✓	✓	P	21	87.5%	High Quality
[76]	✓	✓	✓	✓	✓	✓	✓	✓	X	✓	✓	P	21	87.5%	High Quality
[77]	✓	✓	✓	✓	✓	✓	✓	✓	X	✓	✓	P	21	87.5%	High Quality
[78]	✓	✓	✓	✓	✓	✓	✓	✓	✓	✓	✓	P	23	95.8%	High Quality
[15]	✓	✓	X	✓	✓	✓	P	✓	X	✓	P	P	17	70.8%	Moderate Quality
[79]	✓	✓	✓	✓	✓	✓	✓	✓	X	✓	✓	P	21	87.5%	High Quality
[17]	✓	✓	✓	✓	✓	✓	P	✓	X	✓	✓	P	20	83.3%	High Quality
[80]	✓	✓	✓	✓	✓	✓	✓	✓	✓	✓	✓	P	23	95.8%	High Quality
[81]	✓	✓	✓	✓	✓	✓	✓	✓	X	✓	✓	P	21	87.5%	High Quality
[82]	✓	✓	✓	✓	✓	✓	✓	✓	X	✓	✓	P	21	87.5%	High Quality
[16]	✓	✓	✓	✓	✓	✓	✓	✓	X	✓	✓	P	21	87.5%	High Quality
[83]	✓	✓	✓	✓	✓	✓	✓	✓	✓	✓	✓	P	23	95.8%	High Quality
[84]	✓	✓	X	✓	✓	✓	P	✓	X	✓	P	P	17	70.8%	Moderate Quality
[85]	✓	✓	✓	✓	✓	✓	✓	✓	X	✓	✓	P	21	87.5%	High Quality
[86]	✓	✓	✓	✓	✓	✓	P	✓	X	✓	✓	P	20	83.3%	High Quality
[87]	✓	✓	X	✓	✓	✓	P	✓	X	✓	P	P	17	70.8%	Moderate Quality
[88]	✓	✓	✓	✓	✓	✓	P	✓	X	✓	✓	P	20	83.3%	High Quality
[89]	✓	✓	✓	✓	✓	✓	✓	✓	X	✓	✓	P	21	87.5%	High Quality
[90]	✓	✓	✓	✓	✓	✓	P	✓	X	✓	✓	P	20	83.3%	High Quality
[91]	✓	✓	X	✓	✓	✓	P	✓	X	✓	P	P	17	70.8%	Moderate Quality
[92]	✓	✓	✓	✓	✓	✓	X	✓	X	✓	✓	P	19	79.1%	Moderate Quality
[93]	✓	✓	✓	✓	✓	✓	✓	✓	✓	✓	✓	P	23	95.8%	High Quality
[94]	✓	✓	✓	✓	✓	✓	P	✓	X	✓	✓	P	20	83.3%	High Quality
[95]	✓	✓	X	✓	✓	✓	✓	✓	X	✓	P	P	18	75.0%	Moderate Quality
[96]	✓	✓	X	✓	✓	✓	P	✓	X	✓	P	P	17	70.8%	Moderate Quality
[97]	✓	✓	✓	✓	✓	✓	✓	✓	X	✓	✓	P	21	87.5%	High Quality
[98]	✓	✓	✓	✓	✓	✓	✓	✓	X	✓	✓	P	21	87.5%	High Quality
[99]	✓	✓	X	✓	✓	✓	✓	✓	X	✓	P	P	18	75.0%	Moderate Quality
[100]	✓	✓	✓	✓	✓	✓	✓	✓	X	✓	✓	P	21	87.5%	High Quality
[101]	✓	✓	X	✓	✓	✓	✓	✓	X	✓	P	P	18	75.0%	Moderate Quality
[102]	✓	✓	✓	✓	✓	✓	P	✓	X	✓	✓	P	20	83.3%	High Quality
[103]	✓	✓	X	✓	✓	✓	✓	✓	X	✓	P	P	18	75.0%	Moderate Quality

**Table 3:** Generative AI models used in data augmentation.

Reference	Generative Data Augmentation Model
[24]	Conditional GAN (CGAN) and Wasserstein GAN (WGAN)
[10]	CGAN
[26]	FedCSCD-GAN and VAE
[28]	TVAE and TCGAN for
[12]	Novel Lethality-GAN (L-GAN)
[29]	VAE
[14]	VAEs and GAN
[13]	Conditional GAN (CGAN)
[32]	Phased GAN (PGAN)
[33]	Deep convolutional GAN (DCGAN)
[34]	GAN
[35]	WGAN
[37]	StyleGAN2
[40]	Two-stage GAN (2s-BUSGAN)
[43]	Autoencoder GAN and VAE
[45]	GAN
[49]	CycleGAN
[50]	Distributed multi-latent code inversion enhanced GAN (dm-GAN)
[52]	GAN
[53]	GAN
[55]	DCGAN
[57]	StyleGAN-XL
[59]	Wide-attention SRGAN (WA-SRGAN)
[61]	Anomaly detection with GAN (AnoGAN)
[62]	Deep convolutional GAN (DCGAN) and cycle adversarial network (CCAN)
[63]	GAN
[70]	K-CGAN
[72]	DCGAN
[76]	GAN and VAEs
[78]	GAN
[79]	5-fold data increase GAN (GAN5)
[82]	GAN-modified wasp swarm algorithm (GANMWSA)
[86]	GAN
[51]	Nuclear atypia scoring semi-supervised GAN (NAS-SGAN)
[91]	Conditional GAN (CGAN)
[93]	Wasserstein Generative Adversarial Network Gradient-Penalty (WGAN-GP)
[94]	GAN
[95]	GAN
[98]	Cross Parametric GAN (CPGAN)
[99]	GAN
[102]	Auxiliary Classifier GAN
[103]	DCGAN

**Table 4:** Generative AI models used in classification.

Reference	Generative Classification Models Used
[25]	RNN in combination with WHO and SDM
[27]	Synthesized Gain Modulated Recurrent Neural Network-based Honey Badger Algorithm (GMRNN-HBA)
[30]	Pooling-based Vision Transformer (PiT), Convolutional Vision Transformer (CvT), CrossFormer, CrossViT, NesT, MaxViT, and Separable Vision Transformer (SepViT)
[31]	Convolutional Transformer
[36]	Enhanced RNN
[39]	Holistic Attention Network (HATNet) transformer
[7]	Hybrid CNN-Transformer network (HCTNet)
[41]	Swim-Transformer
[18]	Transformer-based fine-grained (Breast TransFG)
[47]	Transformers and RNN-LSTM
[48]	Advanced Elman Recurrent Neural Network (AERNN)
[51]	RNN-LSTM-GRU
[54]	Multi-modal Transformer (MMT)
[56]	RNN
[58]	Transformer encoder with multilayer perceptron
[60]	Vision transformer block with VGG19
[64]	Multi-view vision transformers
[19]	Breast Ultra Sound via Vision transformers (BUViTNet)
[65]	Vision transformer
[20]	Vision transformer
[87]	Vision transformer
[67]	Fuzzy Relative-Position-Coding (FRPE) transformer
[68]	HoVer-Transformer
[69]	Semantic-aware transformer (SaTransformer)
[71]	RNN
[73]	Two Swin transformer-based architectures: the moderate teacher model and the lightweight student model
[74]	Transformer
[75]	Swin Transformer
[77]	Transformer–CNN
[44]	Target-biased generative adversarial network (TBGAN)
[15]	RNN and CNN hybrid
[104]	Vision transformer
[17]	Transformer-CNN
[80]	Vision transformer
[81]	Graph transformer
[82]	GAN-modified wasp swarm algorithm (MWSA)
[83]	OGRU-LSTM-BiRNN
[84]	Vision transformer
[85]	Vision transformer
[87]	E-transformer
[88]	RNN-RBN
[89]	Vision transformer
[92]	Convolutional Transformer
[96]	RNN
[97]	Histopathology vision transformer (HistoFormer)
[100]	GRU-RNN
[101]	Breast US transformer

**Table 5:** Generative AI models used in segmentation.

Authors	Generative Models Used
[38]	Transformer-based neural network for breast segmentation (TraBS)
[7]	Hybrid CNN-Transformer network (HCTNet) for breast lesion segmentation
[42]	Conditional Generative Adversarial Network
[46]	Breast Tumor Segmentation cGAN (BTS-cGAN)
[41]	Swim-Transformer
[69]	Semantic-aware transformer (Sa Transformer)
[16]	Spider Monkey-based Recurrent Neural System (SMbRNS)
[96]	RNN

**Table 6:** Summary of all works included in the review.

Authors	Generative Models Used	Method Description	Evaluation Metrics
[43]	GAN and VAE for augmentation	A deep structure dubbed AGAN combines GAN and autoencoder for mammographic picture augmentation. Used CNN for mammographic analysis using autoencoder-GAN pictures.	Accuracy—89.71% Sensitivity—93.54% Specificity—80.58% AUC—94.10%
[14]	GAN and VAE for augmentation	Radiogenomics for breast cancer diagnosis using deepfake and autoencoders. Using GANs to generate synthetic radiogenomic datasets produced high-quality images that improved diagnosis accuracy.	AUC—86.3%
[28]	GAN and VAE for data augmentation	Created an interpretable deep-learning framework using TVAE and TCGAN to generate synthetic breast cancer data for diagnosis and prognosis. Trained TabNet, XGBoost, SVM, LR, MLP, and KNN classifiers using fake breast cancer data.	Accuracy–96.76%
[26]	GAN and VAE for data synthesis	GAN-integrated synthetic data for accuracy, VAEs for differential privacy noise. The FedCSCD-GAN breast cancer classification model used federated learning and GAN optimization.	Accuracy–97% Precision—93% Sensitivity—96% Specificity—96%
[76]	GAN and VAEs for augmentation	Data was generated using GAN and triplet-based VAE (TVAE) to increase sample size and rebuild features and labels. Used KNN, SVM, and MLP classifiers.	Accuracy—82.91%
[24]	GAN for augmentation	Two GAN models, CGAN and WGAN, were trained with Unet and Resnet architectures to reduce speckle noise in breast ultrasound images. The classification task used denoised images to identify breast anomalies.	Accuracy—90.41% Sensitivity—87.94% Specificity—85.86%
[12]	GAN for augmentation	Lethality-GAN (L-GAN) was created from the P-GAN model to predict breast cancer in Ductal Carcinoma In Situ (DCIS) using H&E-stained pictures. A Bayesian network combines multi-modal risk scores from clinicopathological data and tumor-infiltrating lymphocyte characteristics in one scalable model.	Area under curve (AUC)—84.3%
[13]	GAN for augmentation	The paired MRI and multi-omic profiles of 61 breast cancer (BC) patients were merged using Bayesian Tensor Factorization to deconstruct the data into 17 latent characteristics. CGAN model was trained using patients’ side-view MRIs and latent characteristics to create MRI scans for BC patients without MRIs. These generated MRIs were used to train a CNN to predict mutation status.	Precision—84.44% Recall—84.35% F1 score—83.36%
[32]	GAN for augmentation	A PGAN-SSN breast ultrasound image categorization model is proposed. PGAN generates difficult samples, extending the dataset. SSN trains generated samples with tagged data. The network learns critical characteristics better by regularizing the features of problematic samples with a hard sample loss function in SSN.	Accuracy—97.7%
[33]	GAN for augmentation	First trained on mammography pictures from three classes, DCGAN generated high-quality synthetic mammograms. Class-specific mean similarity was obtained by blending synthetic and original images. Statistical validation was then used to evaluate the mammograms’ authenticity and reliability.	-
[34]	GAN for augmentation	Proposed a breast cancer detection algorithm using XGBoost trained on GAN-generated synthetic data.	Accuracy—99.04%
[35]	GAN for augmentation	An upgraded WGAN model addressed cancer gene expression data imbalance, giving a reliable training progress indicator and exploring data properties. To balance data, the WGAN generates minority class samples.	Accuracy—98.33%
[37]	GAN for augmentation	Proposed a StyleGAN2-generating model to create high-quality synthetic mammographic pictures and an anomaly detection method for unsupervised breast cancer detection.	AUC—70% Sensitivity—78% Specificity—52%
[40]	GAN for augmentation	Proposed a two-stage GAN architecture (2s-BUSGAN) for annotating BUS photos using Feature-Matching Loss to increase image quality and a Differential Augmentation Module to boost GAN performance on small datasets.	Classification accuracy—85.7% Segmentation dice score—75%
[45]	GAN for augmentation	GANs with a nonlinear identity block, label smoothing, and a new loss function augment ultrasound. Ultrasound segmentation uses modified U-Net 3+.	Dice Score—95.49% Accuracy—95.67% Precision—95.59% Recall—95.68%
[50]	GAN for augmentation	Distributed multi-latent code inversion improved GAN (dm-GAN) for quick, accurate, and automatic breast picture creation. The dm-GAN generator uses a multi-latent code inverse mapping method to simplify data fitting and improve picture creation accuracy, while a multi-discriminator structure improves discrimination accuracy.	-
[52]	GAN for augmentation	Proposed ROImammoGAN synthesizes ROI-based digital mammograms. The model focuses on architectural distortion, asymmetry, bulk, and microcalcification irregularities to ensure that the training process learns their features and delivers enough photos for each category.	SSIM—80% DSSIM—10% PSNR—277.2% MSE—109.92%
[53]	GAN for augmentation	Presented a GAN data augmentation model that realistically synthesizes and removes mammography lesions. Self-attention and semi-supervised learning components in the U-net-based architecture produce mammography-ready 256 × 256 px outputs.	AUC—84.6%
[55]	GAN for augmentation	Proposed DCGAN to synthesize new training pictures for imbalanced data. CNN and GoogleNet classified original and synthetic photos. To handle unbalanced data, DCGAN hyperparameters were tuned, and GoogleNet input size was adjusted to generate synthetic ultrasound pictures.	Accuracy—91.6% Sensitivity—94.2% Specificity—89.74%
[59]	GAN for augmentation	Expanding Low-Resolution (LR) images and creating Super Resolution (SR) images with wide-attention SRGAN (WA-SRGAN). During training, improved Wasserstein with a gradient penalty stabilized the model. The WA ResNeXt101 (32 × 8d) model classified LR, SR, and High-Resolution pictures.	Accuracy—99.11%
[61]	GAN for augmentation	Proposed DenseNet121-AnoGAN for benign and malignant breast histopathology image classification. AnoGAN unsupervised anomaly detection identified mislabeled patches, while DenseNet recovered multi-layered features from discriminative patches.	Accuracy—99.13% F1 score—99.38%
[62]	GAN for augmentation	Tested DCGAN and CycleGAN (CCAN) for breast cancer classification training data enhancement. Some heuristics increased picture similarity between produced and real images.	Accuracy—73.1%
[63]	GAN for augmentation	Proposed a GAN-based anomaly detection model to identify picture anomalies and calculate anomaly scores.	Sensitivity—89.2% Specificity—90.2%, AUC—93.6%
[70]	GAN for augmentation	Proposed a new K-CGAN method to generate synthetic data and verify its performance using MLP, Random Forest, Logistic Regression, Nearest Neighbor, and XGBoost for classification.	Accuracy—98.5%
[72]	GAN for augmentation	Utilized DCGAN to produce synthetic patches, created a semi-supervised framework for IDC identification utilizing a small amount of labeled data and a large amount of unlabeled data.	Accuracy—85.75% F-Measure—77.3%
[78]	GAN for augmentation	A GAN-based virtual elastography US (V-EUS) reconstruction approach was proposed to map BUS to EUS end-to-end.	AUC—89.0% SSIM—93.9%
[49]	GAN for augmentation	Using CycleGAN to enhance BI-RADS 4A malignant lesions’ data, the Dual-ResNet50 classification model autonomously predicted benign and malignant BI-RADS 3 lesions.	AUC—95% Sensitivity—77.8%
[57]	GAN for augmentationViT for Classification	Applied CNN and ViT architectures to public data. Synthetic data from StyleGAN-XL improved model performance. Used explainable AI (class activation maps, centered bounding boxes) to analyze models.	Accuracy—89% Precision—22% AUC—88% Recall—72%
[79]	GAN for augmentation	A 5-fold GAN (GAN5) was proposed to increase restricted data samples. DNA mutation data was employed for gene selection, and GANs expanded RNA expression data. Classification used Random Forest, DNNs, and 1DCNN.	Accuracy—63% F1 score—62%
[86]	GAN for augmentation	GANs change dataset pictures, while U-Net-based CNN layers retain long-range connections and balance partitioning.	PSNR—189.8% SSIM—68.2%
[90]	GAN for augmentation	NAS-SGAN, a semi-supervised generative adversarial network for breast cancer nuclear atypia scoring, uses a stacked supervised and unsupervised discriminator and feature-matching generator.	Accuracy—98.44% F1 score—97.47% AUC—93.27%
[91]	GAN for augmentation	Created a CGAN to generate realistic mammograms with normal appearances utilizing the opposite mammography as a conditional image, identifying MO cancer in thick breasts.	AUC—77%
[93]	GAN for augmentation	To address breast cancer histopathology data imbalance, examined Wasserstein Generative Adversarial Network with Gradient Penalty (WGAN-GP).	Accuracy—95.40% Sensitivity—96.56% Specificity—94.91%
[94]	GAN for augmentation	Created a mammographic GAN-CNN-ELM model for breast cancer detection. Trained the GAN on the dataset to create synthetic mammograms and constructed an ELM classifier to classify feature values to speed up training.	Accuracy—99.0% Recall—99.23% Precision—87.65%
[95]	GAN for augmentation	Created realistic images using standard data augmentation and GAN to boost classification performance.	Accuracy—94%
[98]	GAN for augmentation	Created breast cancer DCE-MRI features using a cross-parametric GAN.	Accuracy—83.8% AUC—86.6% Specificity—76.2% Sensitivity—93.8% F1 score—83.3%
[102]	GAN for augmentation	Using an Auxiliary Classifier Generative Adversarial Network (ACGAN), generated realistic histopathology pictures and class labels. Classification with a deep convolutional neural network (dCNN) assessed the enhanced data’s effectiveness.	Accuracy—90.15%
[103]	GAN for augmentation	Proposed a DCGAN-based synthetic image generation approach to address breast cancer classification’s labeled data shortage.	Accuracy—87% Sensitivity—85.6% Specificity—86.1% F1 score—88%
[99]	GAN for augmentation and classification	Proposed a GAN-based approach to generate IHC images and classify HER2 expression.	PSNR—215.22% SSIM—54.8%
[44]	GAN for classification	Developed TBGAN, a target-biased GAN for source image style transfer, with a prototype transfer module and class-level maximum mean discrepancy loss to match target pictures.	Accuracy—88.9% Precision—89.0% Recall—87.1% F1 score—88.0%
[82]	GAN for classification	A GAN-modified Wasp Swarm Algorithm was created. Used an LSP to preprocess noisy mammography images.	Accuracy—97.34%
[10]	GAN for data augmentation	Used Unet to extract features from 322 mammograms using CNN and skip connections. Created realistic mammograms using CGAN to reduce class imbalance.	Accuracy—81.51% Precision—80.66% F1 score—80.53%
[46]	GAN for segmentation	Proposed BTS-GAN for MRI breast tumor segmentation using cGAN. For better localization, an encoder-decoder network with skip connections was used, and a parallel dilated convolution (PDC) module captured features of different mass sizes and extracted information from their edges and internal textures.	IoU—77% Dice score—85%
[42]	GAN for synthetic segmentation	Created a cGAN to segment a mammography ROI breast tumor. Although the adversarial network distinguishes between genuine and synthetic segmentations, the generative network builds realistic binary masks by identifying the tumor area.	Accuracy—80%
[29]	VAE for data augmentation	Designed a breast cancer classification architecture employing pectoral muscle removal, VAE data augmentation, and U-Net and variation segmentation.	Accuracy—98.5%
[36]	RNN for classification	Federated learning initially collects breast cancer mammography pictures from impacted individuals. DenseNet architecture is used for feature extraction, and an Enhanced RNN optimized by Hybrid Dragon-Rider Optimization (HDRO) employing the Dragonfly Algorithm (DA) and Red Deer Algorithm (RDA) for accuracy is used for classification.	Accuracy—95% MCC—91%
[51]	RNN for classification	Training and classification used a heterogeneous ensemble model with RNN, LSTM, and GRU base classifiers. A fuzzy ARTMAP model controls node number and classifies.	Accuracy—99.36%
[56]	RNN for classification	Proposed a KerasTuner-optimized deep RNN model for breast cancer diagnosis. The model has an input layer, five hidden layers, five dropout layers, and an output layer with optimized neuron counts and dropout rates in each hidden layer.	Accuracy—94.00% Precision—94.00% Recall—94.00%
[71]	RNN for classification	Used RNN to predict breast cancer.	F1 score—97% Precision—97% Recall—97%
[27]	RNN for classification	GMRNN-HBA detects breast cancer utilizing histology images, which provide comprehensive cancer cell information and help classify disease severity stages.	Accuracy—98.2% Recall—97% Precision—95.8% Specificity—96.8% F1 score—96.1%
[48]	RNN for classification	Proposed a hybrid model using LDA, WHO, and AERNN. WHO reduces features and tunes hyperparameters, LDA selects features, and optimized AERNN classifies.	Precision—98.51% Recall—98.65% Accuracy—97.88% F1 score—98.32%
[15]	RNN for classification	Proposed an RNN-CNN hybrid technique to classify genomic data into cancer classifications. Sandwich stacking with pre-trained VGG16 and VGG19 models extracted bottleneck characteristics.	Accuracy—97.8%
[83]	RNN for classification	Used a stacked OGRU-LSTM-BiRNN model and multivariate data analysis to find breast cancer progression indicators in Raman spectral characteristics.	Accuracy—96.64%
[88]	RNN for classification	RNN-RBN classification and XGBoost-embedded genetic algorithm feature selection improved breast cancer diagnosis accuracy.	Accuracy—97.748% Precision—97.40% F1 score—96.9%
[100]	RNN for classification	Using deep learning, created a GRU-RNN model with optimal parameter changes and disease-relevant obstructive characteristics.	Accuracy—95.61% Precision—97.22% Recall—95.89% F1 score—95%
[96]	RNN for classification and segmentation	Proposed a hybrid model using EfficientNetV2s and RNN for classification and U-Net with an attention layer for breast histology image segmentation.	Precision—99% Recall—99% F1 score—99%
[16]	RNN for segmentation	Proposed a Spider Monkey-based Recurrent Neural System (SMbRNS) to improve segmentation and early breast cancer cell prediction.	Accuracy—99.82% Precision—99.12%
[25]	RNN in combination with WHO and SDM for classification	Contrast-Enhanced Images are segmented by LS-CED. The SPM-R-FCN algorithm detects cancer cell nuclei. Adaptive thresholding classifies tumor cells (TCs) as benign, malignant, or normal. For reliable breast cancer detection and malignancy classification, the SDM-WHO-RNN classifier receives relevant information from categorized TCs.	Accuracy—97.9% Sensitivity—98.98% Specificity—96.51%
[7]	Transformer for classification and segmentation	Improved ultrasound breast lesion segmentation with a Hybrid CNN-Transformer network (HCTNet). In the encoder, Transformer Encoder Blocks (TEBlocks) capture global contextual information and extract features with CNNs. To eliminate semantic inconsistencies with the encoder, the decoder uses a Spatial-wise Cross Attention (SCA) module.	Accuracy—97.41% Recall—97.33% Precision—97.14%
[104]	Transformer for classification	Proposed a benign/malignant mammographic image classification system using EfficientNet B0, Focal Loss, and ViT-base.	Accuracy—100% AUC—100%
[74]	Transformer for classification	Analyzed DBT stack neighbors using transformer architecture. The proposed method was compared to a three-dimensional convolution architecture and a two-dimensional model that analyzes each region separately.	AUC—91% Sensitivity—87.7 Specificity—86.4%
[54]	Transformer for classification	A Multi-modal Transformer (MMT) was created to identify cancer patients and estimate future cancer risk in cancer-free persons. MMT tracks temporal tissue changes by comparing current tests to historical images and combines multimodal data utilizing self-attention.	AUROC—94.3% AUPRC—51.8%
[60]	Transformer for classification	Detected breast cancer using a vision transformer (ViT) block with VGG19 and a parallel module to extract global and local features. Fusing retrieved features with cross-attention integrated major semantic variances.	Accuracy—81%
[64]	Transformer for classification	Captured long-range correlations between patient mammograms using multi-view vision transformers. Local transformer blocks use two-view (CC/MLO) and two-side (right/left) pictures to learn patch relationships in four mammography images. Concatenating and feeding outputs from different views and sides into global transformer blocks helped learn cross-view relationships.	AUC—81.8%
[19]	Transformer for classification	Introduced ViT-based breast ultrasound image classification framework BUViTNet. Transfer learning for breast ultrasound classification was preceded by multistage transfer learning utilizing ImageNet and cancer cell imaging datasets.	AUC—96. 8% MCC—96.1% Kappa—95.9%
[65]	Transformer for classification	Proposed ETECADx by combining CNN ensemble transfer learning with self-attention-based ViT. The transformer encoder used binary classification and multi-classification to predict breast cancer.	Accuracy—98.58% for binary class Accuracy—97.87% for multi-class
[68]	Transformer for classification	Released GDPH&SYSUCC, a big breast lesion classification dataset. A BUS-anatomical HoVer-Transformer is proposed. The HoVer-Trans block extracts horizontal and vertical spatial information. Integrating convolutional layers between transformer stages allows horizontal and vertical image feature fusion and inductive bias.	AUC—92.4% Accuracy—89.3% Specificity—83.6% Sensitivity—92.6%
[73]	Transformer for classification	Proposed moderate teacher and lightweight student Swin Transformer architectures. Used transfer learning to train the teacher model and knowledge distillation (KD) to train the student model. Increased validation accuracy and KD loss reduction using SARSA reinforcement learning.	Accuracy—98.71% for student model Accuracy—98.91% for teacher model
[75]	Transformer for classification	Tested an ensemble of Swin Transformers for two-class (benign vs. malignant) and eight-class (four benign and four malignant subtypes) classification.	Accuracy—96.0% for eight class Accuracy—99.6% for binary class
[20]	Transformer for classification	Built a deep learning mammographic breast cancer detection model combining transfer learning and vision transformers with augmentation-based class balancing.	Accuracy—100%
[31]	Transformer for classification	Proposed a hybrid multi-class classification model using depth-wise separable convolutional networks and transformers to capture local and global data. Implemented patch merging to simplify transformer self-attention modules.	Accuracy—98.32%
[39]	Transformer for classification	Holistic Attention Network (HATNet), a self-attention-based model for end-to-end breast biopsy picture classification. Segmented biopsy images into patches using a bag-of-words technique and self-attention to encode inter-word and inter-bag interactions.	Accuracy—71% F1 score—70% Sensitivity—71% Specificity—90% ROC-AUC—90%
[18]	Transformer for classification	Proposed transformer-based fine-grained breast cancer classification model Breast TransFG Plus. Added a component selection module plus for key information extraction utilizing matrix addition and a double-head dual-stream classification structure. Data augmentation and balanced sampling were used on the training set.	Accuracy—99.39% Precision—99.18% Recall—99.59%
[58]	Transformer for classification	For breast cancer classification, used ResNet for deep feature extraction and a Transformer Encoder (TE) and MLP.	Accuracy—100% for binary class Accuracy—95.8% for multiclass
[66]	Transformer for classification	Developed ViT-based breast cancer categorization utilizing supervised and self-supervised learning. Used adaptive token sampling (ATS) to select important image tokens dynamically for ViT. Created a bespoke consistency training technique to integrate supervised, SSL, and image augmentation.	Accuracy—98.12% Precision—98.17% Recall—98.65% F1 score—98.41%
[67]	Transformer for classification	Breast ultrasound (BUS) picture classification using fuzzy relative-position-coding (FRPE) transformer. BUS pictures were separated into non-overlapping patches, encoded using fuzzy relative-position encoding, processed through a transformer encoder, then identified as benign or malignant using an MLP.	Accuracy—90.52% Precision—90.52% Recall—90.52% F1 score—90.52%
[17]	Transformer for classification	A CNN-Transformer hybrid model for breast cancer detection and classification. Used a depthwise convolution-based hierarchical backbone to extract deep features and an EFB to aggregate multi-level features. Global feature extraction with Scale-Denoising Attention (SDA) transformer.	Accuracy—96.56% Sensitivity—95.55% Specificity—97.88% AUC—93.13%
[80]	Transformer for classification	The proposed method used NasNetLarge, MobileNetV2, InceptionV3, DenseNet, and ViT to classify mammograms as normal, benign, or malignant.	Accuracy—99%
[81]	Transformer for classification	Improved graph transformer layer node featured embedding with local connectivity aggregation.	Accuracy—92%
[84]	Transformer for classification	Test ViT-based transfer learning for breast image classification.	Accuracy—95% AUC—95% F1 score—95%
[85]	Transformer for classification	Presented the Bootstrap Your Own Latent (BYOL) self-supervised learning (SSL) technique for diagnostic mammography image processing.	-
[44]	Transformer for classification	E-transformer-based model for breast cancer lymph node metastatic prediction addressed fine-grained classification.	AUC—96.43% Accuracy—96.81% F1 score—96 69%
[89]	Transformer for classification	Created vision transformer-based breast mammogram interpretation CAD system.	Accuracy—96.48%
[92]	Transformer for classification	Conv-Transformer, ConvMixer, and CoatNet hybrid models were tested to help pathologists identify breast cancers in histopathological pictures at various magnifications.	Accuracy—99%
[41]	Transformer for segmentation Transformer for classification	Integrating Swin Transformer (ST) into a CNN-based U-Net, the BTS-ST network improved global modeling. A Spatial Interaction Block (SIB) was created to encode spatial knowledge in the Swin Transformer block using pixel-level correlations.	Classification F1 score—93. 7% Precision—98% Recall—89.8% AUC—94.4% Segmentation F1 score—90.8% Ji—82.9% AUC—97.8% HD95—68.59%
[38]	Transformer for segmentation	TraBS, a transformer-based neural network for breast segmentation, was developed and tested utilizing multi-institutional MRI data. It was compared to the popular nnUNet CNN model.	Dice score—91.6% ASSD on internal—54.8% ASSD on external— 58.4%
[69]	Transformer for segmentation and classification	Established the Semantic-aware Transformer (SaTransformer) for breast cancer segmentation and classification. Semantic interaction between classification and segmentation tasks improved feature learning and reduced memory and computational complexity.	Accuracy—97.97% Precision—98.20% DSC—86.34% JSC—83.12%
[47]	Transformers and RNN for classification	Used BERT pre-training of Image Transformers (BEiT) for feature encoding to combine vision transformers (ViT) with transfer learning. The encoded features were categorized by RNN-LSTM.	Accuracy—97.6%
[30]	Transformers for classification	For histopathology image classification, evaluated transformer-based designs PiT, CvT, CrossFormer, CrossViT, NesT, MaxViT, and SepViT. Compared ViT-integrated models for accuracy and generalization.	Accuracy—60% to 100% Precision—86% Recall—80% F1 score—85%
[97]	Transformers for classification	Developed HistoFormer, a high-performance vision transformer-based model for ductal carcinoma subtype diagnosis using stain-normalized histopathology images.	Accuracy—88% Precision—93% Recall—95% F1 score—94%
[101]	Transformers for classification	Introduced the transformer-based BreastUS Transformer for breast ultrasound image classification.	Accuracy—97% Recall—97% Precision—97% F1 score—96%

**Table 7:** Datasets used by the studies in the review.

Data Sets Utilized	Description	Accessibility	Authors	Frequency
AIDPATH Dataset (https://aidpath.eu) accessed on 25 September 2024	Includes 50 WSIs of an invasive tumor at 40× magnification. Their testing is limited to invasive tumor classification due to inadequate annotations.	Public	[30]	1
The Breast Cancer Histology Images (BACH) Dataset [104]	The 15th International Conference on Image Analysis and Recognition co-hosted BACH. The 400 photos are classified as benign, in situ, invasive, and normal.	Public	[30,31,73,81,96,97]	6
Breast Carcinoma Subtyping (BRACS) Dataset [105]	IRCCS Fondazione Pascale, ICAR of the National Research Council (CNR), and IBM Research-Zurich collaborated on it. The dataset contains 547 whole slide images (WSIs) from 189 patients and 4537 ROIs from 387 WSIs from 151 patients.	Public	[30,81]	2
Breast Cancer Histopathological Image Classification (BreakHis) dataset [106]	BreakHis, released in 2016, contains 7909 histological pictures from 82 breast cancer patients. The dataset divides tumors into benign and malignant. Phyllodes Tumor (PT), Tubular Adenoma (TA), Fibroadenoma (F), and Adenosis (A) are benign tumors, whereas Papillary Carcinoma (PC), Ductal Carcinoma (DC), Lobular Carcinoma (LC), and Mucinous Carcinoma (MC) are malignant.	Public	[29,44,47,59,61,66,73,75,92,99,102]	11
Breast biopsy dataset [107]	The breast biopsy dataset has 240 H&E-stained WSIs. Benign without atypia (includes non-proliferative and proliferative), atypia, DCIS, and invasive cancer are its four classifications. The dataset has 422 ROIs.	Public	[39]	1
Breast Invasive Carcinoma (BRCA) data set (http://www.cancergenome.nih.gov/) accessed on 29 September 2024	A dataset of 19,738 genes with mRNA and DNA mutations from diverse cancer types.	Public	[35,79]	2
BUSI dataset [108]	Collection from Baheya Women’s Hospital for Early Detection and Treatment of Cancer (Cairo, Egypt) utilizing LOGIQ E9 and E9 Agile ultrasound devices. The BUSI dataset comprises 780 photos from 600 female patients aged 25–75, averaging 500 × 500 pixels. It has 437 benign, 210 malignant, and 133 normal instances.	Public	[7,19,24,40,47,55,66,67,68,69]	12
Cancer Metastases in Lymph Nodes (Camelyon) 16 dataset (https://camelyon16.grand-challenge.org) accessed on 25 September 2024	The 2016 research challenge dataset includes 400 WSIs with a resolution of 218,000 × 95,000 pixels. The collection comprises normal and cancerous pictures.	Private	[59]	1
Cancer Center of Sun Yat-sen University dataset [109]	Imaged at 7.1 MHz with the HDI 5000 SonoCT System’s L12-5 50 mm Broadband Linear Array. The collection consists of 320 photos from patients aged 46.6 ± 14.2 years, comprising 160 benign and 160 malignant tumors. The picture size was adjusted to 128 by 128 pixels.	Private	[7]	1
Collected dataset	Multiple acquisition systems were used by Beijing’s China-Japan Friendship Hospital to assemble the dataset in 2018. The 920 photos include 470 benign and 450 cancerous instances. Pathology from puncture biopsy findings determines benign or malignant status.	Public	[40]	1
Databiox dataset	Databiox contains histopathology microscope pictures of IDC-identified patients between 2014 and 2019 in Iran by Poursina Hakim’s research center for grade categorization. The dataset has 922 pictures.	Private	[27]	1
Dataset from the Hospital of the University of Pennsylvania and the Cancer Institute of New Jersey (http://www.andrewjanowczyk.com/use-case-6-invasive-ductal-carcinoma-idc-segmentation) accessed on 25 October 2024	The collection contains digitized breast cancer histopathology slides from 162 IDC patients.	Private	[72]	1
DBUI dataset	Contains 437 benign and 210 malignant breast nodule mask samples.	Public	[32,101]	2
DDSM dataset [110]	Digital mammograms from 2500 instances with 43 volumes were collected at the University of South Florida. Each patient’s MLO and CC views are included.	Public	[17,20,33,36,41,42,43,50,58,82,84,94,95,103,104]	15
DUKE dataset [111]	The Duke Hospital dataset comprises 922 biopsy-confirmed invasive breast cancer patients from 2000 to 2014. General Electric or Siemens 1.5 Tesla or 3.0 Tesla scanners captured the photos.	Public	[38]	1
Exome dataset [112]	Has 4181 sample versions and 88 characteristics.	Public	[76]	1
GDPH&SYSUCC dataset [68]	The ultrasounds came from two medical centers: the Department of Ultrasound, Guangdong Provincial People’s Hospital (GDPH), Guangzhou, China, and the Department of Ultrasound, Sun Yat-sen University Cancer Center (SYSUCC), Guangzhou. The collection contains 2405 BUS images: 1519 cancer from 676 patients and 886 benign from 526 individuals.	Public	[68]	1
Hospital Sant Joan de Reus dataset [42]	The ground truth binary masks of 300 malignant tumors (123 Luminal-A, 107 Luminal-B, 33 Her-2, and 37 Basal-like) were acquired by radiologists.	Private	[42]	1
INBreast dataset [113]	The INBreast dataset has 410 photos from 115 patients, including 360 images from 90 women afflicted in both breasts, collected from April 2008 to July 2010 at the University Hospital (Centro Hospital de São João [CHSJ], Breast Center, Porto, Portugal).	Public	[42,60,65,84,89,95]	6
Kaggle dataset (https://www.kaggle.com/datasets/paultimothymooney/breast-histopathology-images) accessed on 25 September 2024	The collection includes 5547 50 × 50 × 3 breast histology pictures, with 2788 labelled as invasive ductal carcinoma (IDC) and 2759 as non-IDC.	Public	[16,62,93]	3
Mendeley breast ultrasound dataset [114]	Brazil-collected pictures from women aged 30–70 are classified as benign (100) or malignant (150).	Public	[19]	1
METABRIC dataset (https://www.cbioportal.org/study/summary?id=brca_metabric) accessed on 25 November 2024	From clinical CNA and gene expression datasets, the METABRIC dataset comprises 1980 patient records.	Public	[77]	1
MIAS dataset [115]	The collection comprises 312 left and right breast mammography pictures from 161 people. The photos are 1024 × 1024.	Public	[10,29,52,80,82,84,86,95]	8
NYU Breast Cancer Diagnosis Multi-modal Dataset	A dataset of 1,353,521 FFDM, DBT, and ultrasound tests from 297,751 NYU Langone Health patients between 2010 and 2020.	Public	[54]	1
Optimam Mammography Image Database, UK [116]	The collection contains 8282 photos with malignant lesions, 1287 with benign lesions, and 16,887 with normal lesions from a large UK screening sample.	Public	[53]	1
Pathology databases at Oxford University (Oxford, UK) and Singapore General Hospital (SGH), Singapore [12]	This multi-institutional cohort has 133 cases, 45 of which are breast cancer-related.	Private	[12]	1
Patients’ data from 15 centers in Shangai [78]	The collection contains 4488 examples classed as 1861 invasive, 214 in situ, 151 malignant, 1520 fibroadenoma, and 834 benign instances.	Private	[78]	1
Radiological Society of North America (RSNA) dataset [117]	It includes 11,913 exams, 486 of which are cancer cases.	Public	[57]	1
RIDE dataset (https://www.cancerimagingarchive.net/collection/tcga-brca/) accessed on 25 September 2024	The dataset includes 500 ground truth segmented DCE-MRI images from various patients.	Public	[46]	1
Southwest Hospital dataset [49]	The 2015–2021 dataset comprises 742 individuals with 847 lesion pictures, including 418 BI-RADS 6 benign, 220 malignancies, and 209 4A tumors.	Private	[49]	1
Tangshan Hospital dataset [49]	The 2021 dataset includes 354 patients with 428 lesion pictures, including 7 BI-RADS 3 malignancies and 421 benign cases.	Private	[49]	1
TCIA datasets [118]	The collection includes 187 three-dimensional DCE-MRI images from 61 individuals.	Private	[13]	1
The Cancer Genome Atlas (TCGA) dataset (http://www.cancergenome.nih.gov/) accessed on 5 September 2024	Multiomic data from 754 patients comprises CNV score, gene expression, and DNA methylation.	Public	[13,14]	2
UC Irvine dataset	Has 561 occurrences and 31 characteristics.	Private	[71]	1
UDIAT-Centre Diagnostic, Corporacio Parc Tauli, Sabadell, Spain [119]	A Siemens ACUSON Sequoia C512 system with a 17L5HD linear array transducer (8.5 MHz) was used to gather data from the UDIAT Diagnostic Center of Parc Taulí Corporation in Sabadell. The collection consists of 163 BUS pictures from various women, averaging 760 by 570 pixels, including 53 cancerous and 110 benign instances.	Public	[7,24,68,69]	4
UKA dataset [38]	UKA comprises 9751 breast MRI scans from 5086 women from 2010 to 2019 at the University Hospital Aachen in Germany.	Private	[38]	1
Wisconsin dataset (https://www.kaggle.com/uciml/breast-cancer-wisconsin-data) accessed on 19 September 2024	The 1995 Wisconsin Hospitals University dataset comprises 569 samples from 32 individuals, with 62.74% benign and 37.26% malignant.	Public	[26,28,48,56,70,100]	6
University Medical Center, Hospital, Nursultan, Kazakhstan data set [85]	There are 1571 DICOM mammography pictures in the dataset.	Private	[85]	1
Imaging Center of Henan Provincial People’s Hospital data set [120]	The dataset comprises 391 clinical cases from 28 to 85-year-olds, including 169 lymph node metastases and 222 without.	Private	[44]	1

**Table 8:** Evaluation Metrics Utilized by the studies in the review.

Evaluation Metric	Description	Equation	References	Frequency
Accuracy	Measures the proportion of correct results (both true positives and true negatives) out of the total number of cases evaluated.	$\frac{TP+TN}{TP+TN+FP+FN}$	[7,10,13,14,15,16,17,19,20,31,32,33,36,39,40,42,43,44,46,47,49,50,51,52,53,55,56,59,63,64,65,66,67,68,72,73,74,75,76,77,78,79,81,82,84,85,86,87,88,89,90,91,92,95,96,97,98,100,101,102,103,105,106,108,109,111]	66
Recall/Sensitivity	Sensitivity is a numerical value that reflects the ability of a method to accurately identify patients who have breast cancer.	$\frac{TP}{TP+FN}$	[7,10,13,14,15,16,19,32,36,38,45,46,48,50,51,52,53,56,57,63,64,69,74,75,76,80,81,82,84,85,86,101,102,104,105,106,108,109,111]	39
F1 Score	Put together precision and sensitivity into a single metric	$\frac{2\times precision\times sensitivity}{precision+sensitivity}$	[8,13,26,30,39,41,44,48,61,66,67,70,71,72,84,87,88,90,96,97,98,100,101,103]	24
Precision	Demonstrates how effectively a method correctly classifies cases.	$\frac{TP}{TP+FP}$	[8,16,18,25,26,27,30,40,41,44,45,48,56,57,66,67,70,71,88,94,96,97,100,101]	24
Specificity	Complements sensitivity by emphasizing the model’s ability to accurately identify negative cases, thereby reducing false positives.	1−FP	[7,17,24,25,27,37,41,55,63,68,74,98,103]	13
AUC-ROC	It is used to assess a classifier’s ability to differentiate between classes.	The area under the ROC curve	[14,17,19,28,37,39,41,43,49,54,55,57,63,64,68,70,71,74,77,78,84,87,91,93,98]	25
Matthew’s Correlation Coefficient (MCC) score	Considers true and false positives and negatives, providing a balanced measure even for imbalanced datasets.	$\frac{TN\times TP-FN\times FP}{\surd (TP+FP)(TP+FN)(TN+FP)(TN+FN)}$	[19,36,71]	3
Kappa Score	Evaluates the agreement between model predictions and ground truth data, ensuring reliable and consistent diagnostic capabilities.	$\frac{2(TN\times TP-FN\times FP)}{\left(TP+FP)(FP+TN)+(TP+FN)(FN+TN\right)}$	[19,71]	2
Dice Similarity Coefficient (DSC) Score	Measures the degree of overlap between the predicted labels and the ground truth labels.	$\frac{2|x\cap y|}{\left|x\right|+|y|}$	[38,40,45,46,69]	5
Intersection Over Union (IOU) or Jaccard Similarity Coefficient (JSC) score	Measures the overlap between predicted and actual regions of interest	$\frac{\left|x\cap y\right|}{\left|x\cup y\right|}$	[41,46,69]	3
Structural similarity indexing method (SSIM)	Used to evaluate the similarity between two images	$\frac{\left(2\mu_{real}\mu_{virtual}+C_{1})(2\sigma_{real,virtual}+C_{2}\right)}{\left(\mu_{real}^{2}+\mu_{virtual}^{2}+C_{1}\right)(\sigma_{real}^{2}+\sigma_{virtual}^{2}+C_{2})}$	[52,78,86,99]	4
Structural dissimilarity (DSSIM)	Evaluates how much one image deviates from another in terms of both intensity and structural information	$\frac{1-SSIM(x,y)}{2}$	[52]	1
Peak Signal-to-Noise Ratio (PSNR)	Quantifies the similarity between original image and processed image by measuring the fidelity of the reconstructed data.		[86,99]	2

## Data Availability

No new data were created or analyzed in this study.
